# Amphiphilic Copolymer-Lipid Chimeric Nanosystems as DNA Vectors

**DOI:** 10.3390/polym14224901

**Published:** 2022-11-13

**Authors:** Varvara Chrysostomou, Aleksander Foryś, Barbara Trzebicka, Costas Demetzos, Stergios Pispas

**Affiliations:** 1 Section of Pharmaceutical Technology, Department of Pharmacy, School of Health Sciences, National and Kapodistrian University of Athens, Panepistimioupolis Zografou, 15771 Athens, Greece; 2 Theoretical and Physical Chemistry Institute, National Hellenic Research Foundation, 48 Vassileos Constantinou Avenue, 11635 Athens, Greece; 3 Centre of Polymer and Carbon Materials, Polish Academy of Sciences, 34 ul. M. Curie-Skłodowskiej, 41-819 Zabrze, Poland

**Keywords:** non-viral vectors, gene delivery, cationic lipids, amphiphilic random copolymers, chimeric/hybrid lipoplexes

## Abstract

Lipid-polymer chimeric (hybrid) nanosystems are promising platforms for the design of effective gene delivery vectors. In this regard, we developed DNA nanocarriers comprised of a novel poly[(stearyl methacrylate-co-oligo(ethylene glycol) methyl ether methacrylate] [P(SMA-co-OEGMA)] amphiphilic random copolymer, the cationic 1,2-dioleoyl-3-(trimethylammonium) propane (DOTAP), and the zwitterionic L-α-phosphatidylcholine, hydrogenated soybean (soy) (HSPC) lipids. Chimeric HSPC:DOTAP:P[(SMA-co-OEGMA)] nanosystems, and pure lipid nanosystems as reference, were prepared in several molar ratios of the components. The colloidal dispersions obtained presented well-defined physicochemical characteristics and were further utilized for the formation of lipoplexes with a model DNA of linear topology containing 113 base pairs. Nanosized complexes were formed through the electrostatic interaction of the cationic lipid and phosphate groups of DNA, as observed by dynamic, static, and electrophoretic light scattering techniques. Ultraviolet–visible (UV–Vis) and fluorescence spectroscopy disclosed the strong binding affinity of the chimeric and also the pure lipid nanosystems to DNA. Colloidally stable chimeric/lipid complexes were formed, whose physicochemical characteristics depend on the N/P ratio and on the molar ratio of the building components. Cryogenic transmission electron microscopy (Cryo-TEM) revealed the formation of nanosystems with vesicular morphology. The results suggest the successful fabrication of these novel chimeric nanosystems with well-defined physicochemical characteristics, which can form stable lipoplexes.

## 1. Introduction

Recently, gene therapy has gained prominence as one of the most promising therapeutic approaches for the treatment of genetic-based diseases [1]. The implementation of gene therapy involves the delivery of therapeutic nucleic acid-based medicines into cells to correct a cellular dysfunction or to provide a new cellular function in order to treat or prevent disorders [2,3]. A determinant factor for the successful application of this promising therapeutic strategy is the use of efficient carriers for the effective nucleic acid transfer and cellular uptake [4]. These gene delivery carriers, also known as vectors, are broadly classified into viral and non-viral vectors [5]. Viral vectors including viruses such as adenovirus, adeno-associated virus, lentivirus, retrovirus, herpes simplex virus, poxvirus, etc. have been employed for the delivery of therapeutic agents. Moreover, viral vectors are considered an effective means for the delivery of nucleic acids and have shown success in vivo and in clinical trials [6,7]. However, viral vectors are associated with serious safety issues, including acute immune response and insertional mutagenesis [4,5,6]. Although non-viral vectors are less efficient, their advantages regarding low biosafety risk, potential for large-scale production, controllable chemical structure, wide material source, multifunctionality, and no capacity limits in gene encapsulation have brought them at the forefront of gene delivery research [4,5,6,7,8].

Non-viral gene carriers based on cationic lipids or polymers or even more interesting a combination of these are now among the most promising technologies for gene therapy and therapeutics [9]. In particular, the cationic lipid-based liposomes represent one of the most intensively studied and clinically advanced platforms in the field of the non-viral vectors [10,11,12]. Cationic liposomal formulations utilized for gene delivery are frequently composed of a neutrally charged lipid and a cationic lipid. Neutral lipids, often called helper lipids, are components for a cationic liposome formulation in which they play an assistant role in stabilizing bilayer membranes [13,14]. Furthermore, the combination of a cationic lipid with a neutral lipid has conferred improved efficiency to the cationic lipids [14]. Cationic lipids are amphiphilic molecules consisting of a positive charged headgroup, covalently bound through a linker to a hydrophobic tail [15]. They can be easily synthesized and are considered as one of the most versatile tools for the delivery of nucleic acids and other therapeutic molecules [15]. The positively charged head groups are amines, quaternary ammoniums, guanidinium, or amino acids [10,16] which can electrostatically interact with the anionic phosphate groups of nucleic acids, leading to the formation of complexes containing condensed nucleic acids, namely lipoplexes [15,16,17]. The most frequent group is quaternary ammonium, due to its permanent positive charge that provides strong interaction with the nucleic acids and enhanced solubility in aqueous environments [10,16].

Lipoplexes protect the genetic material from enzymatic degradation, increase the stability of the vector, and interact with the cell membrane through electrostatic interactions [18]. Thus, the lipoplexes are typically formed with a slight excess of positive charge to permit their interaction with the negatively charged cell surface [19,20]. The transfection efficiency and stability of lipoplexes are strongly affected by a variety of formulation factors, including the lipid to nucleic acid charge ratio, the lipoplex size, the surface charge, and environmental conditions such as the ionic strength, temperature, and pH of the medium [16,21,22]. Compared to viral vectors, lipoplexes are formed spontaneously, their preparation is simple and cost-effective, and they do not present the risk of the insertion of genetic material into the host’s genome [23]. However, lipoplexes are associated with cytotoxicity effects due to the permanent positive charge of the cationic lipids, which has become one of the main bottlenecks for their application, with their clinical utilization remaining a challenge [23,24,25]. Therefore, scientific research has been focused on developing delivery systems based on innovative nanomaterials that are able to overcome these hurdles.

The advances on the science of nanotechnology have greatly benefited progress in the exploration of new non-viral nanocarriers. The combination of different in nature materials can change their individual properties and generate hybrid nanostructures with new features. Particularly, the combination of lipid-based and polymer-based nanosystems is considered an innovative approach for biomedical applications [26,27,28]. A new generation of nanosystems has been created by harnessing the advantages of lipids, such as biomimetic nature and biocompatibility, with the advantages of polymers, such as versatility in chemical structure, chemical functionalities, and response to external stimuli [28,29]. The design of lipid and polymer-based nanoparticles as non-viral vectors, which can protect nucleic acids and ensure their targeted delivery, and also to promote controlled release and enhanced cellular uptake, has gained significant attention [30]. Regarding the lipoplexes, the combination of cationic lipids with polymers is more favorable, thus it can prevent the binding affinity of non-specific proteins to the lipoplexes and can improve circulatory half-lives [31,32,33]. In general, the synergistic effect of these two pillar classes of materials can lead to the generation of multifunctional nanocarriers capable of simultaneously delivering, in a single platform, different therapeutic compounds including hydrophobic drugs, nucleic acids, proteins/peptides, and diagnostic agents, e.g., magnetic nanoparticles and dyes for bioimaging, as a combinational therapeutic approach [7,29,33,34].

In this study, novel hybrid/chimeric (i.e., nanosystems composed of lipids and copolymers) [35] nanocarriers are developed for nucleic acid delivery utilizing the cationic 1,2-dioleoyl-3-(trimethylammonium) propane (DOTAP) lipid, which is one of the most widely used and efficient lipid transfection agents for gene delivery applications [36]. DOTAP consists of a quaternary ammonium salt as the cationic head group [16], which facilitates the spontaneous electrostatic interaction with nucleic acids forming lipoplexes, as well as the binding of the resulting lipoplexes to the negatively charged components of the cell membrane [37]. Different DOTAP-based lipoplexes have entered preclinical and clinical trials [16]. For instance, lipoplexes composed of DOTAP and cholesterol (DOTAP:chol) have been clinically investigated for the treatment of various diseases including several types of cancer [23]. As in most cationic lipid formulations, DOTAP, due to the increased density of positive charges on liposome surface, exhibits inefficient gene transfer and cannot be used alone [37,38]. Hence, to improve its gene transfection capabilities and to provide colloidal stability, DOTAP is utilized in combination with other neutral/helper lipids to self-assemble into cationic liposomes [15,16,39,40]. In this direction, we utilized the neutral lipid of L-α-phosphatidylcholine, hydrogenated soybean (soy) (HSPC), which has high melting temperature and can be used to construct highly stable liposomes and therefore to provide stability [40].

In the next step, employing the reversible addition fragmentation chain transfer (RAFT) polymerization, we synthesized the novel amphiphilic random copolymer of poly[(stearyl methacrylate-co-oligo(ethylene glycol) methyl ether methacrylate] [P(SMA-co-OEGMA)]. RAFT polymerization gives well-defined polymers with predetermined molecular characteristics for their implementation in gene delivery applications [41,42]. Moreover, the facile preparation of block and random copolymers through RAFT, permits scale-up production, which is important for the design of a gene delivery system [43]. The amphiphilic copolymer consists of two segments, the hydrophobic SMA and the hydrophilic OEGMA. The (P)SMA is considered a super hydrophobic polymer, possessing a long alkyl side chain of 18 CH_2_ which can form crystalline domains [44,45,46]. The long hydrophobic chain in the SMA segment serves as an anchor for the incorporation of the copolymer inside the lipid bilayers, providing stability to the liposomal formulation [47,48]. On the other hand, (P)OEGMA is composed of a hydrophobic main chain and grafted hydrophilic side oligo(ethylene glycol) chains [43,49]. The toxicity of the cationic lipids remains an important issue, because of their high positive charge density. Accordingly, DOTAP also exhibits a cytotoxicity effect due to its quaternary amine headgroup [25,50]. The PEG-shielding strategy has been adopted in the cationic nanocarriers to mask the excessive positive charges [39]. Similar to a PEG-strategy, we utilized the non-ionic (P)OEGMA with average M_n_ = 475 g∙mol^−1^ and nine ethylene glycol repeated units as an alternative shielding agent, which displays similar properties to PEG [51,52], in an effort to mask the positive charges of DOTAP and therefore to reduce cytotoxicity effects and provide stealth properties, biocompatibility, and colloidal stability to the lipoplexes.

In this regard, we developed the novel HSPC:DOTAP:P[(SMA-co-OEGMA)] hybrid/chimeric liposomal nanosystem and we explored its potential to bind DNA and form lipoplexes. Chimeric nanosystems of HSPC:DOTAP:P[(SMA-co-OEGMA)] were prepared by the thin film hydration method, in several molar ratios of the copolymer and lipid components, including the ratios of 9:1:0.05, 9:1:0.1, 7:3:0.05, and 7:3:0.1. Furthermore, pure liposomal formulations of HSPC:DOTAP lipids were also prepared in molar ratios of 9:1 and 7:3 and were used as reference systems. The colloidal dispersions were examined by light scattering techniques for the determination of their physicochemical characteristics, such as their size and size distribution, morphology, and surface charge. Afterward, we investigated the ability of the chimeric nanosystems and also of the references pure liposomes, to electrostatically interact with nucleic acids, utilizing a double stranded model DNA from salmon testes of linear topology and length of 113 base pairs (bp). Nanosized complexes formed through the electrostatic interaction of the cationic quaternary amino group of the DOTAP component with the phosphate group of DNA, in a wide range of N/P ratios (nitrogen (N) of amine group of cationic lipid over phosphate (P) groups of DNA). The interaction process and the formed complexes were investigated using light scattering (dynamic, static and electrophoretic), fluorescence and UV–Vis spectroscopy and Cryo-TEM microscopy, in order to comprehensively study their physicochemical and morphological characteristics. In addition, the stability of the chimeric/lipid lipoplexes in increasing ionic strength, as a simulation of the physiological conditions of the biological fluids, was also evaluated by dynamic light scattering.

## 2. Materials and Methods

### 2.1. Materials

For the synthesis of the random copolymer, the monomer stearyl methacrylate (SMA, ≥89.5%) and the oligomer oligo(ethylene glycol) methyl ether methacrylate (OEGMA), with average M_n_ = 475 g/mol and 9 ethylene glycol units, were purchased from Sigma-Aldrich (Athens, Greece). The SMA and OEGMA monomers were purified by passing through columns packed with hydroquinone monomethyl ether (MEHQ) and butylated hydroxytoluene (BHT) inhibitor removers before the polymerization process. The MEHQ and BHT inhibitor removers, the chain transfer agent 4-cyano-4-(phenylcarbonothioylthio)pentanoic acid (CPAD), the radical initiator 2,2-azobis(isobutyronitrile) (AIBN), 1,4-dioxane (99.8%), tetrahydrofuran (THF, ≥99.9%), chloroform (99.9%), and deuterated chloroform (CDCl_3_) (99.9%) were also obtained from Sigma-Aldrich (Athens, Greece) and used as received, except 1,4-dioxane, which was dried over molecular sieves before use. Moreover, AIBN was purified by recrystallization from methanol and subsequently used as a solution in 1,4-dioxane. Dialysis tubing membranes (MEMBRA-CEL^®^) from regenerated cellulose of MWCO 3500 and a diameter of 22 mm were obtained by SERVA (Heidelberg, Germany).

**Scheme 1:**
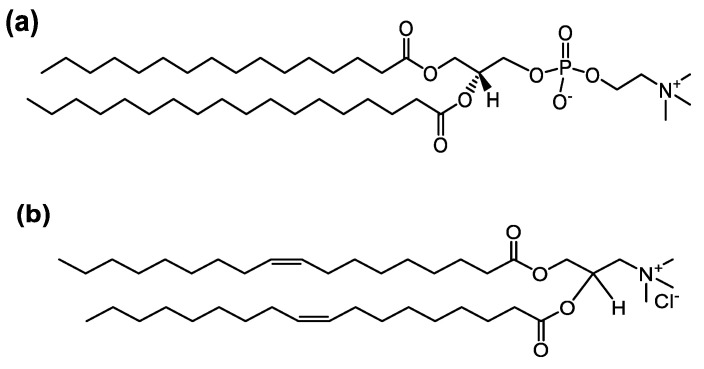
Chemical structures of (**a**) L-α-phosphatidylcholine, hydrogenated soybean (soy) (HSPC) and (**b**) 1,2-dioleoyl-3-(trimethylammonium) propane (chloride salt) (DOTAP) lipids.

The lipids L-α-phosphatidylcholine, hydrogenated soybean (soy) (HSPC) (Scheme 1a) and 1,2-dioleoyl-3-(trimethylammonium) propane (chloride salt) (DOTAP) (Scheme 1b) were purchased from Avanti Polar Lipids Inc. (Alabaster, AL, USA) and used without further purification.

For the interaction of the lipid and chimeric nanosystems with nucleic acids, linear double stranded deoxyribonucleic acid sodium salt from salmon sperm of 113 base pairs length was purchased from Acros. Ethidium bromide (EtBr) dye for DNA quenching fluorescent assay and sodium chloride (≥99.0%), which was utilized for the preparation of NaCl solutions of 1 M, were received from Sigma-Aldrich (Athens, Greece). All the solutions were prepared using sterile water for injection (DEMO SA., Athens, Greece).

### 2.2. Synthesis of the Amphiphilic Random Copolymer

**Scheme 2:**
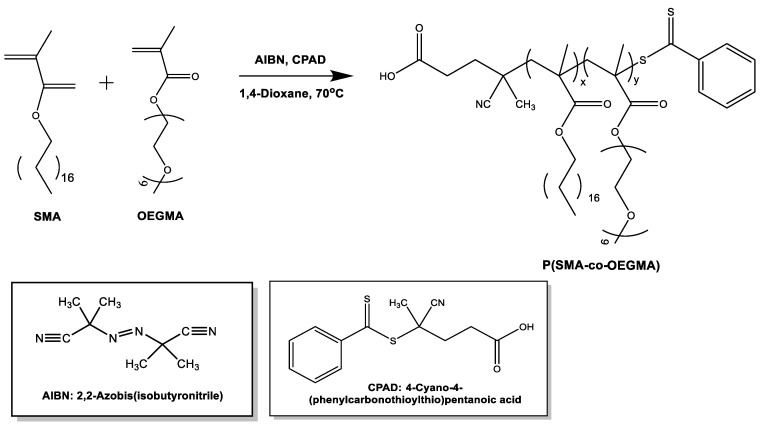
Synthetic route for the synthesis of P(SMA-co-OEGMA) copolymer via RAFT polymerization.

The synthesis of the novel amphiphilic random P(SMA-co-OEGMA) copolymer was achieved by employing reversible addition-fragmentation chain transfer (RAFT) polymerization. The copolymer was synthesized following a one-step synthetic procedure as described in detail below. The purified monomers SMA (0.6 g, 1.77 mmol) and OEGMA (1.4 g, 2.95 mmol), the CPAD (0.05 g, 0.20 mmol) chain transfer agent, and the AIBN (0.0065 g, 0.04 mmol) radical initiator were placed in a round bottom flask equipped with a magnetic stirrer and were dissolved in 10 mL of 1,4-dioxane (20 wt.% monomer solution) under stirring. The used CTA (CPAD) to initiator (AIBN) ratio ([CTA]_0_/[I]_0_) was 5:1. The flask was sealed with a rubber septum. The reaction solution was degassed by high purity nitrogen gas bubbling for 20 min and then immersed in a preheated oil bath at 70 °C for 24 h. The reaction was quenched by freezing the solution at −20 °C for 30 min and exposing it to air. Unreacted monomers or other impurities and 1,4-dioxane were removed by dialysis against deionized H_2_O for 3 days with three exchanges per day. The purified copolymer was dried under vacuum oven for 48 h at room temperature and collected at >98% yield. The followed synthetic polymerization route and the chemical structure of the copolymer are depicted in Scheme 2.

### 2.3. Size Exclusion Chromatography (SEC)

A Waters size exclusion chromatography (SEC) instrument (Waters Corporation, Milford, MA, USA) was utilized for the determination of the molar mass (M_w_), molar mass distributions, and dispersity index (M_w_/M_n_) of the synthesized P(SMA-co-OEGMA) copolymer. The chromatography system is equipped with a Waters 1515 isocratic pump (Waters Corporation, Milford, MA, USA), a set of three µ-Styragel mixed pore separation columns (pore size 10^2^–10^6^ Å), and a Waters 2414 differential refractive index detector (equilibrated at 40 °C) (Waters Corporation, Milford, MA, USA). The measurements and data analysis were conducted using the Breeze software (Waters Corporation, Milford, MA, USA). Tetrahydrofuran (THF) containing 5% *v/v* trimethylamine was the mobile phase, at a flow rate of 1 mL/min and temperature set at 30 °C. The calibration curve was set by utilizing linear polystyrene standards with average molecular mass in the range of 1200–152,000 g·mol^−1^ and narrow molecular mass distributions. The copolymer was dissolved in the mobile phase and measured at concentration of 1 mg mL^−1^.

### 2.4. Proton Nuclear Magnetic Resonance (^1^H NMR) Spectroscopy

^1^H-NMR spectroscopy was implemented to confirm the chemical structure and to determine the mass composition (%wt.) of the synthesized copolymer. The spectrum was recorded using a Bruker AC 300 MHz FT-NMR spectrometer (Bruker, Billerica, MA, USA) and deuterated chloroform (CDCl_3_) as the solvent. The chemical shifts are reported in parts per million (ppm) relative to tetramethylsilane (TMS) as the internal standard in CDCl_3_.

^1^H-NMR spectral peaks of P(SMA-co-OEGMA) copolymer (300 MHz, CDCl_3_: 7.26 ppm, δ): 4.08 (peak c_2_: 2H, -COOC***H_2_***(CH_2_)_16_CH_3_-), 3.89 (peak c_1_: 2H, -(C=O)OC***H_2_***CH_2_O), 3.64 (peak f: 36H, -(C***H_2_***C***H_2_***O)_9_CH_3_-), 3.37 (peak g: 3H, -(CH_2_CH_2_O)_9_C***H_3_***-), 1.60 (peak b: 4H, -C***H_2_***C-), 1.25 (peak f: 32H, -CH_2_(C***H_2_***)_16_CH_3_-), 0.88 (peak a: 6H, -CH_2_CC***H_3_***, peak e: 3H, -CH_2_ (CH_2_)_16_C***H_3_***-).

### 2.5. Fourier-Transform Infrared (FT-IR) Spectroscopy

Fourier-transform infrared (FT-IR) spectroscopy in the form of ATR-FTIR was also employed to verify the chemical structure of the synthesized copolymer. The mid-infrared measurement was conducted at room temperature, in the spectral range of 5000–550 cm^−1^, using a Fourier transform instrument (Bruker Equinox 55, Bruker Optics GmbH, Ettlingen, Germany) equipped with a single bounce attenuated total reflectance (ATR) diamond accessory (Dura-Samp1IR II by SensIR Technologies, Chapel Hill, NC, USA). The polymer sample was measured in the solid state and the spectrum was recorded after 64 scans with a resolution of 4 cm^−1^.

ATR-FTIR spectral peaks of P(SMA-co-OEGMA), *v* (cm^−1^), (s: stretching, b: bending): (CH_2_): 2922 (s), 2854 (s) and 1454 (b), (C=O): 1728 (s), (C-O-C): 1105 (s).

### 2.6. Preparation of Pure Lipid and Chimeric Nanocarriers

Conventional liposomal nanocarriers of HSPC and DOTAP lipids and mixed/chimeric liposomes composed of HSPC, DOTAP lipids, and the amphiphilic random P(SMA-co-OEGMA) copolymer were prepared in different molar ratios by mixing the appropriate amounts of each component and following the thin film hydration method. Specifically, suitable amounts of HSPC, DOTAP, and P(SMA-co-OEGMA) were added in a round bottom flask and dissolved in chloroform. Subsequently, the mixture was placed under vacuum and heat using a rotary evaporator (Rotavapor R-114, Buchi, Switzerland), at 45 °C aqueous bath for 60 min, until the evaporation of the solvent and the formation of a thin film layer on the wall of the flask. The film was maintained under vacuum for 2 h and then in a desiccator for at least 24 h to remove possible traces of solvent. Afterward, the film was hydrated with water for injection by slowly spinning the round bottom flask in a water bath for 60 min, at a temperature 10 °C above the main phase transition of the main lipid component (*T*_m_ ≈ 52 °C for HSPC [35], *T*_m_ ≈ −1 °C for DOTAP [53]) to ensure complete hydration. The resultant structures were subjected to two 1-min sonication cycles (amplitude 70%, cycle 0.5 s) using a probe sonicator (UP 200 S, DrHielsher GmbH, Berlin, Germany) interrupted by a 3 min resting period in order to prevent sample overheating. Then, the obtained nanostructures were allowed to anneal for 30 min. The same procedure was followed for the preparation of both pure and chimeric liposomal formulations. Namely, two HSPC:DOTAP pure liposomal formulations with molar ratios 9:1 and 7:3 and four HSPC:DOTAP:P(SMA-co-OEGMA) chimeric nanosystems with molar ratios 9:1:0.05, 9:1:0.1, 7:3:0.05 and 7:3:0.1 were produced. The colloidal concentration was 10 mg∙mL^−1^ in all the prepared dispersions. However, the physicochemical studies were performed on diluted samples with concentration of 0.5 mg∙mL^−1^.

### 2.7. Preparation of Pure and Chimeric Lipoplexes

Lipoplexes were formed through the electrostatic interaction between the cationic DOTAP and the DNA. The procedure followed for generating the lipoplexes includes as a first step the preparation of pure/chimeric liposomes (0.5 mg∙mL^−1^, same concentration for all liposomal formulations) and DNA 113 bp (concentration ranging from 0.018 to 0.065 mg∙mL^−1^) stock aqueous solutions. The utilized concentration of DNA stock solutions was calculated taking into consideration the moles of the cationic DOTAP in each liposomal formulation. Hence, DNA stock solutions with concentration of 0.018 mg∙mL^−1^ were prepared in the case of liposomes with lower molar ratio in DOTAP, while stock solutions with concentration of 0.065 mg∙mL^−1^ were prepared for liposomes with higher molar ratio in DOTAP. The preparation of the lipoplexes was achieved by mixing stock solutions of each liposomal formulation with different amounts of the relevant DNA stock solutions, under gentle stirring, at room temperature and neutral pH. The mixing amounts of the solutions are based upon calculations related to the molar ratio of nitrogen (N) from the positively charged quaternary amine group of DOTAP to the phosphate (P) from the negatively charged phosphate groups of DNA backbone, referred to as the Nitrogen-to-Phosphate ratio (N/P). The amounts of liposomes and DNA solutions were selected to give lipoplexes with N/P ratios ranging from 0.5 to 8. The formed lipoplexes were studied after allowing them to stand overnight at ambient temperature for equilibration.

### 2.8. Ultraviolet–Visible (UV–Vis) Spectroscopy

The interaction of the pure and chimeric liposomes with the DNA at different N/P ratios was explored by ultraviolet–visible (UV–Vis) spectroscopy. The absorption spectra of the lipoplexes were recorded on a Perkin Elmer (Lambda 19) UV–Vis–NIR spectrophotometer (Waltham, MA, USA) in the wavelength range of 200–600 nm. The recorded UV–Vis spectra of the lipoplexes as well as those of the pure and chimeric liposomes are presented in Figures S1 and S2 (Supplementary Materials), respectively.

### 2.9. Fluorescence Spectroscopy-Ethidium Bromide Quenching Assay

A standard fluorescence quenching assay based upon ethidium bromide (EtBr) exclusion was utilized to determine whether the pure/chimeric liposomes can bind DNA. Ethidium bromide was added in aqueous DNA stock solutions (0.01 mg∙mL^−1^) at a molar ratio, EtBr = P/4, where P corresponds to the molar concentration of DNA phosphate groups. The DNA solutions containing EtBr were left overnight to equilibrate and to ensure the complete intercalation of EtBr into the free DNA. Subsequently, the labeled DNA-EtBr solutions were titrated using concentrated aqueous solutions of liposomes (0.5 mg⋅mL^−1^) in the range of N/P ratio 0 (neat DNA-EtBr) solution to 8. After the titration, the solutions at the studied N/P ratios were equilibrated for 15 min at 25 °C, before the operation of fluorescence spectroscopy measurements. The measurements were conducted on a Fluorolog-3 Jobin Yvon-Spex spectrofluorometer (model GL3–21). The excitation wavelength used for the recorded spectra was at 535 nm, while the emission was monitored at 600 nm. [54,55].

### 2.10. Light Scattering

The physicochemical characteristics regarding the size (hydrodynamic radius, R_h_), the size distribution (Polydispersity index, PDI) the morphology, and the surface charge (zeta-potential, Z_p_) of all the prepared pure/chimeric formulations and the formed pure/chimeric lipoplexes were determined by light scattering techniques including dynamic (DLS), static (SLS) and electrophoretic (ELS) light scattering, respectively. Prior to light scattering measurements, the solutions were filtered through 0.45 μm hydrophilic PVDF syringe filters (Membrane Solutions, Auburn, WA, USA) to remove large aggregates and dust particles.

DLS measurements were implemented on an ALV/CGS-3 compact goniometer system (obtained from ALV GmbH, Langen, Hessen, Germany), equipped with a cylindrical JDS Uniphase 22 mW He–Ne laser (ALV GmbH, Langen, Hessen, Germany), operating at 632.8 nm. The system was interfaced with an ALV-5000/EPP multi-τ digital correlator (ALV GmbH, Langen, Hessen, Germany) with 288 channels and an ALV/LSE-5003 light scattering electronics (ALV GmbH, Langen, Hessen, Germany) unit for stepper motor drive and limit switch control. Moreover, a Polyscience 9102A12E bath circulator (Polyscience, Illinois, USA) was utilized to regulate the temperature inside the measuring cell. Toluene was used as the calibration standard. The measurements were implemented on the angular range of 45° to 135°, at 25 °C. The scattered light intensity was simultaneously monitored. The autocorrelation functions were recorded five times for each angle and averaged. The obtained correlation functions were fitted and analyzed by the cumulants method and the CONTIN algorithm. The apparent hydrodynamic radius, R_h_, was calculated using the Stokes–Einstein equation. The presented data of R_h_, PDI, and scattered light intensity (I) correspond to measurements at 90°.

Static light scattering (SLS) measurements were performed on the same instrument at 25 °C, in the angular range of 30–150°, at 10° intervals and using toluene as the calibration standard. SLS measurements were treated by the Zimm second order plot to estimate the radius of gyration R_g_ and therefore the R_g_/R_ho_ ratio, after extrapolation to zero angle (R_ho_). The R_g_/R_h_ ratio provides useful information on the morphology and the shape of the nanoparticles.

Electrophoretic light scattering (ELS) measurements were also performed at 25 °C using a Nano Zeta Sizer (Malvern Instruments Ltd., Worcestershire, UK) composed of a 4 mW solid-state He–Ne laser, operating at 633 nm and at a fixed backscattering angle of 173°. Zeta-potential values were determined using the Henry approximation of the Smoluchowski equation. The recorded zeta-potential values were averages of 50 scans, with an error smaller than ±2 mV.

### 2.11. Cryogenic Transmission Electron Microscopy (Cryo-TEM)

Cryogenic Transmission Electron Microscopy (cryo-TEM) images were obtained using a Tecnai F20 X TWIN microscope (FEI Company, Hillsboro, OR, USA) equipped with a field emission gun, operating at an acceleration voltage of 200 kV. Images were recorded on the Gatan Rio 16 CMOS 4 k camera (Gatan Inc., Pleasanton, CA, USA) and processed with Gatan Microscopy Suite (GMS) software (Gatan Inc., Pleasanton, CA, USA). Specimen preparation was done by vitrification of the aqueous solutions on grids with holey carbon film (Quantifoil R 2/2; Quantifoil Micro Tools GmbH, Großlöbichau, Germany). Prior to use, the grids were activated for 15 s in oxygen plasma using a Femto plasma cleaner (Diener Electronic, Ebhausen, Germany). Cryo-samples were prepared by applying a droplet (3 μL) of the suspension to the grid, blotting with filter paper and immediate freezing in liquid ethane using a fully automated blotting device Vitrobot Mark IV (Thermo Fisher Scientific, Waltham, MA, USA). After preparation, the vitrified specimens were kept under liquid nitrogen until they were inserted into a cryo-TEM-holder Gatan 626 (Gatan Inc., Pleasanton, CA, USA) and analyzed in the TEM at −178 °C.

## 3. Results and Discussion

### 3.1. Synthesis and Molecular Characterization of Random Copolymer

The synthesis of the novel amphiphilic random poly[(stearyl methacrylate)-co-oligo(ethylene glycol)methyl ether methacrylate] P[(SMA-co-OEGMA)] copolymer was accomplished following a one-step RAFT polymerization procedure. In this regard, the 4-cyano-4-(phenylcarbonothioylthio)pentanoic acid (CPAD) was selected as the CTA, which is reactive and compatible with methacrylate monomers [49,56,57,58]. The applied polymerization conditions were also chosen based on literature data [49,58,59].

**Figure 1:**
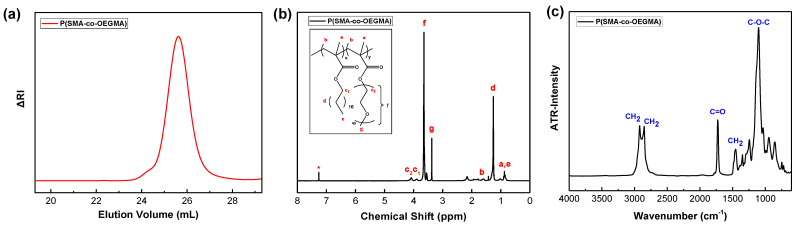
(**a**) SEC chromatogram of the synthesized P(SMA-co-OEGMA) copolymer in THF/5% *v/v* Et_3_N. (**b**) ^1^H-NMR spectrum of P(SMA-co-OEGMA) copolymer in CDCl_3_. The peak at 7.26 ppm (*) is assigned to the solvent protons. (**c**) ATR-FTIR spectrum of P(SMA-co-OEGMA) copolymer in solid state.

The copolymer was molecularly characterized by means of SEC chromatography and ^1^H-NMR and ATR-FTIR spectroscopies (Figure 1). The chromatogram in Figure 1a, as obtained by SEC, depicts narrow, monomodal, and symmetric molar mass distribution. The molecular mass (M_w_) of the copolymer and the polydispersity index (M_w_/M_n_) were also determined by SEC, with values of 12,240 g∙mol^−1^ and 1.11 respectively. The resulting molecular mass is close to the stoichiometry and the polydispersity index is narrow.

^1^H-NMR spectrum (Figure 1b) verified the expected chemical structure of the synthesized P(SMA-co-OEGMA) copolymer. The signals at δ 1.26 ppm corresponding to the methylene -CH_2_ protons of the SMA alkyl side chain (peak d, 32H, (CH_2_)_16_) [46] and the one at δ 3.64 ppm to the -CH_2_ protons of the OEGMA ethylene glycol side chain (peak f, 36H, -CH_2_CH_2_O)_9_) [60] were utilized to quantify copolymer composition, which was found to be 38%wt. for the SMA segment and 62%wt. for the OEGMA one.

Along with ^1^H-NMR, ATR-FTIR spectroscopy was also utilized. The ATR-FTIR spectrum of P(SMA-co-OEGMA) copolymer (Figure 1c) contains all the major chemical groups of which the copolymer is comprised, indicating the expected chemical structure. Specifically, the absorption bands observed at 2922 cm^−1^ and 2854 cm^−1^ correspond to C-H stretching vibrations of the -CH_2_ groups of SMA and OEGMA side groups, while the band at 1454 cm^−1^ corresponds to bending vibrations also of the -CH_2_ groups [61]. The strong stretching vibration band at 1722 cm^−1^ is ascribed to the C=O carbonyl of the ester group of both segments [61]. The broad band at 1105 cm^−1^ corresponds to C–O–C stretching vibrations of the ether group of OEGMA. [61,62]. Furthermore, the absence of vibrations of the C=C bond in the ATR-FTIR spectrum proves the successful polymerization at high monomer conversion.

Τhe obtained results from SEC, ^1^H-NMR, and ATR-FTIR indicate that the followed synthetic route and polymerization conditions worked satisfactorily. The followed RAFT methodology provided control of the molecular characteristics, which is an important issue in the design of copolymers for gene delivery.

### 3.2. Physicochemical Characterization of Pure and Chimeric Liposomes

**Figure 2:**
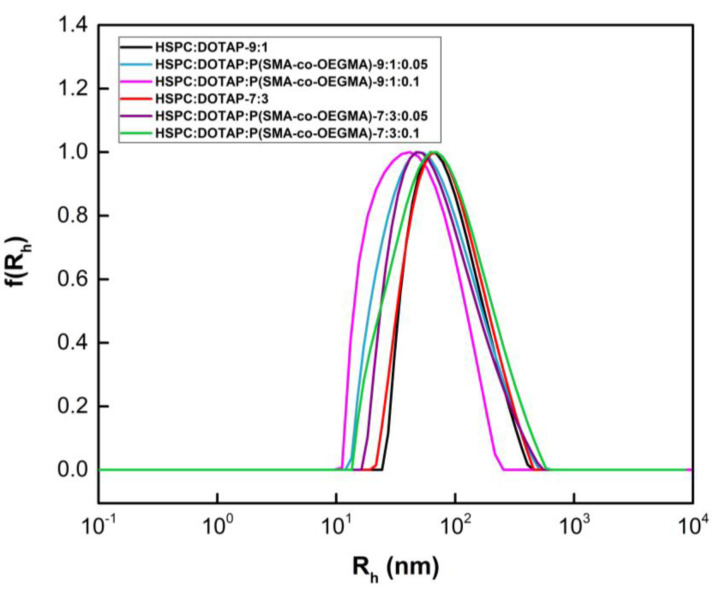
Size distributions from CONTIN analysis of HSPC:DOTAP 9:1 and 7:3 liposomes, HSPC:DOTAP:P(SMA-co-OEGMA)-9:1:0.05, 9:1:0.1, 7:3:0.5, and 7:3:0.1 chimeric liposomes in H_2_O, C=0.5 mg·mL^−1^ at a scattering angle θ = 90°.

The physicochemical characteristics of the developed nanosystems in aqueous solutions were examined by light scattering techniques. Dynamic light scattering (DLS) was employed to determine the size of the formed nanostructures by estimating the R_h_ and the size polydispersity index (PDI). The determined values are presented in Table 1. The hydrodynamic size distribution plots from CONTIN analysis in Figure 2 for pure HSPC:DOTAP liposomes and chimeric HSPC:DOTAP:P(SMA-co-OEGMA) liposomes revealed that both types of liposomes self-assemble into a single, uniform, relatively narrow, and well-defined nanoparticle population.

All liposomal formulations presented sizes with hydrodynamic radius less than 100 nm and polydispersity index, PDI ≥ 0.45. However, according to the R_h_ values of Table 1 and the size distribution plots of Figure 2, it is evident that the incorporation of the amphiphilic random copolymer significantly reduced the size of the chimeric liposomes compared to that of the pure liposomes. This reduction can be attributed to the presence of hydrophobic and steric effects due to the anchoring of the long alkyl chains of the SMA segment into the lipid bilayers.

The zeta-potential values as determined by electrophoretic light scattering (ELS) are also listed in Table 1. Both types of liposomes exhibit positive zeta-potential values, with the liposomes comprised of higher molar ratio of DOTAP showing more positive absolute values. The increase of DOTAP ratio caused a great increase of the surface charge particularly in the HSPC:DOTAP-7:3 liposomes. It is evident that the higher ratio of the quaternary ammonium head group in the DOTAP lipid contributed to the strong positive charge of the liposomes. On the other hand, the incorporation of the amphiphilic copolymer led to a significant decrease of the zeta-potential values. This decrease was found to depend on copolymer molar ratio. The chimeric liposomes with molar ratio 0.1 presented lower zeta-potential values compared to those with 0.05. This reduction is assigned to the non-ionic OEGMA moieties, which are located on the outer liposomal surface and promote a shielding effect of the liposomes. Hence, these findings revealed that the incorporation of the amphiphilic copolymer and particularly the hydrophilic OEGMA chains efficiently reduced the strong cationic surface charge, which is important to prevent cytotoxicity effects and to produce effective gene delivery nanocarriers.

Static light scattering was utilized in order to extract information regarding the shape and morphology of the liposomal nanostructures, by determining the R_g_/R_h_ ratio. The calculated values are summarized in Table 1. The liposomal nanoassemblies presented R_g_/R_h_ values ranging from 0.80 to 1.11, indicating their assembly into vesicular morphologies [63,64]. Furthermore, in the case of the chimeric liposomes, the incorporation of the copolymer caused a slight increase of the R_g_/R_h_ ratio, with the values remaining in the range of vesicles morphology.

Conclusively, light scattering techniques demonstrate the formation of liposomes with well-defined physicochemical characteristics, which are highly dependent on the molar ratio of each component which the liposomes consist of. Moreover, the obtained size values of the liposomes are in a satisfactory nanoscale range for gene nanocarriers. The efficient reduction of the high positive charges due to the shielding effect of OEGMA segment signifies that such segments are a useful tool for the control of the nanocarrier surface charge.

### 3.3. Ethidium Bromide Quenching Assay by Fluoresence Spectroscopy

To elucidate whether the pure and chimeric liposomes can bind DNA, fluorescence spectroscopy was employed by studying the quenching of ethidium bromide (EtBr) probe. Ethidium bromide is a cationic fluorescent probe, which intercalates between the adjacent base pairs of DNA double helix and exhibits a strong fluorescent intensity [65,66]. The electrostatic interaction between the positively charged liposomes and the DNA results to the exclusion of EtBr from DNA double helix to solution aqueous environment, accompanied by an evident decay of its fluorescence intensity. Therefore, fluorescence quenching of EtBr provides an indirect way to determine the binding affinity between the cationic liposomes and DNA and thus ascertain the formation of lipoplexes.

**Figure 3:**
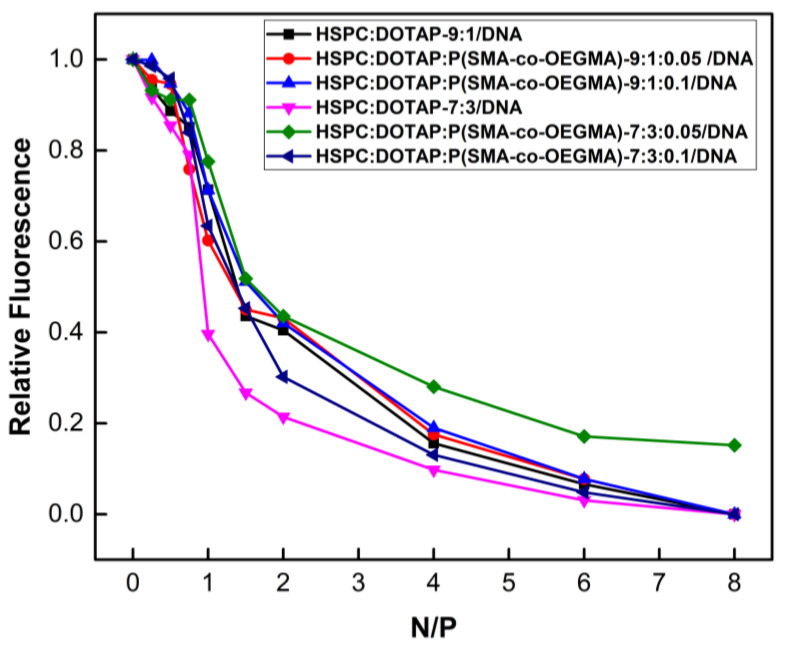
Ethidium bromide quenching in lipoplexes formed from the interaction of HSPC:DOTAP and HSPC:DOTAP:P(SMA-co-OEGMA) liposomes with the DNA of 113 bp, utilizing liposomes of different molar ratios.

In this regard, the quenching of EtBr was examined in a range of N/P = 0 to N/P = 8, by titration of the liposomal solutions to the DNA-EtBr solution. The recorded spectra demonstrating the reduction in the fluorescence intensity of the intercalated EtBr upon the progressive addition of the cationic pure/chimeric liposomes are presented in Figure S3. Figure 3 depicts the typical curves of the relative fluorescence intensity of EtBr as a function of the N/P ratio, for the HSPC:DOTAP/DNA and HSPC:DOTAP:P(SMA-co-OEGMA)/DNA complexes. The curves exhibit a well-pronounced and gradual decrease of the fluorescence intensity for all the studied liposomal nanosystems.

Concerning the interaction of the pure HSPC:DOTAP liposomes with the DNA, the formed HSPC:DOTAP-7:3/DNA and the HSPC:DOTAP-9:1/DNA complexes present a similar trend in the decrease of the fluorescence intensity of the EtBr until the ratio N/P = 0.75. However, the decrease of the relative fluorescence intensity for the HSPC:DOTAP-7:3/DNA complexes at the ratio N/P = 1 and until the N/P = 8 is sharper, indicating the faster rate of the EtBr displacement and thus the better binding affinity of the HSPC:DOTAP-7:3 liposomes to DNA. The stronger binding affinity of the HSPC:DOTAP-7:3 nanocarriers was expected due to the higher molar content of these liposomes in DOTAP. Nevertheless, similar to HSPC:DOTAP 7:3 liposomes, the HSPC:DOTAP-9:1 liposomes also present sufficient complexation ability with DNA, due to the fact that at the ratio N/P = 8 the relative intensity was found equal to zero, denoting that the liposomes displaced efficiently the whole amount of the intercalated EtBr from the DNA double helix.

The HSPC:DOTAP:P(SMA-co-OEGMA)/DNA complexes formed by the chimeric liposomes and the DNA displayed similar behavior in EtBr exclusion. All the chimeric nanosystems exhibited the same displacement rate of EtBr with the relative fluorescent intensity reaching zero at N/P = 8, except for HSPC:DOTAP:P(SMA-co-OEGMA)-7:3:0.05 liposomes in which the relative fluorescence reached 0.15 at the same N/P ratio. The shielding effect of OEGMA is reflected in the rate of decrease of the relative fluorescence intensity, which in the case of the chimeric liposomes is somehow lower compared to the reduction rate for the HSPC:DOTAP-7:3 and HSPC:DOTAP-9:1 pure liposomes. Particularly, the HSPC:DOTAP:P(SMA-co-OEGMA) chimeric liposomes with molar ratio 9:1:0.05 and 9:1:0.1 present similar trend in the decrease of the relative fluorescence intensity, with the HSPC:DOTAP:P(SMA-co-OEGMA-9:1:0.05 liposomes displaying a slightly faster decrease from N/P = 0.75 to N/P = 8, in comparison with the 9:1:0.1 system. However, similar behavior is not observed in the case of the chimeric liposomes with molar ratios 7:3:0.05 and 7:3:0.1, with the latter presenting the more abrupt decrease of EtBr relative fluorescence intensity in comparison with all the chimeric nanosystems studied. This fact was expected due to the high molar ratio of DOTAP compared to the chimeric nanosystems with molar ratio 9:1:0.05 and 9:1:0.1. Nevertheless, all the chimeric nanosystems presented a strong binding affinity to the DNA of 113 bp, which was confirmed by the complete exclusion of EtBr from the DNA double helix.

Summarizing, the EtBr quenching assay provided better proof for lipoplex formation. The presence of the OEGMA chains probably affected the exclusion rate of EtBr; however, it is significant that at the ratio N/P = 8, total exclusion of the EtBr was observed for most of the investigated liposomal nanosystems, indicating their strong binding affinity to DNA. Hence, the liposomes can efficiently interact with the DNA, exhibiting strong binding affinity, which is a required parameter of an efficient gene nanocarrier.

### 3.4. Lipoplexes Characterization by Light Scattering

The physicochemical properties of the lipoplexes formed by the complexation of HSPC:DOTAP and HPSC:DOTAP:P(SMA-co-OEGMA) liposomes with the DNA of 113 bp, were further evaluated by light scattering techniques. The determination of their size and surface charge is essential for their efficient implementation as non-viral gene delivery nanocarriers since these parameters can affect the biological performance of the lipoplexes.

The lipoplexes were prepared and studied in a wide range of N/P ratios (N/P = 0.25 to N/P = 8), including ratios with an excess of DOTAP positive charges and an excess of DNA negatively charges, in an attempt to gain a better view on the complexation process and to find the ratios with the preferable complexation efficiency and colloidal stability. All the dispersions appeared colloidally stable by the naked eye. However, lipoplexes formed either by pure or chimeric liposomes presented partial precipitation at the ratio N/P = 1, in which ratio the charges of DOTAP are stoichiometrically equal to those of DNA. The precipitation of the lipoplexes probably occurred due to charge neutralization, which resulted to the decrease of their solubility and therefore their precipitation. Furthermore, it is noteworthy to mention that the partially precipitated chimeric lipoplexes appeared more stable by naked eye compared to the pure lipoplexes. This observation indicates that the presence of the non-ionic OEGMA segment as well as the long alkyl chain of the SMA segment probably contributed to the colloidal stability of the lipoplexes and prevented their total collapse. Light scattering measurements of the precipitated lipoplexes were performed on the supernatant of the solutions. The precipitation region is denoted in the presented data of the light scattering plots; however, the results for the ratio N/P = 1 are not taken into consideration.

**Figure 4:**
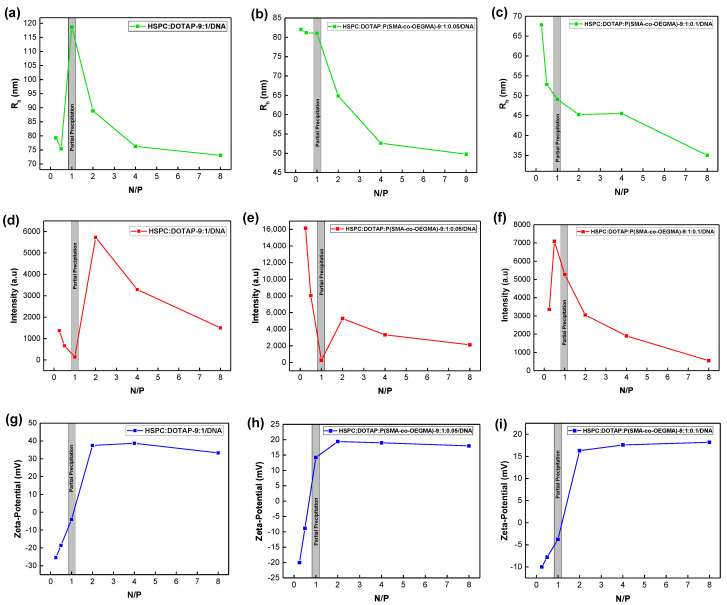
Variations of hydrodynamic radius (R_h_) with N/P ratio for (**a**) HSPC:DOTAP-9:1/DNA, (**b**) HSPC:DOTAP-9:1:0.05/DNA and (**c**) HSPC:DOTAP-9:1:0.1/DNA lipoplexes. Variations of scattered light intensity with N/P ratio for (**d**) HSPC:DOTAP-9:1/DNA, (**e**) HSPC:DOTAP-9:1:0.05/DNA, and (**f**) HSPC:DOTAP-9:1:0.1/DNA lipoplexes. Variations of zeta-potential with N/P ratio for (**g**) HSPC:DOTAP-9:1/DNA, (**h**) HSPC:DOTAP-9:1:0.05/DNA and (**i**) HSPC:DOTAP-9:1:0.1/DNA lipoplexes.

Light scattering findings regarding the size, intensity, and zeta potential of the lipoplexes as a function of the N/P ratio are presented in Figure 4 for the HSPC:DOTAP-9:1/DNA, HSPC:DOTAP:P(SMA-co-OEGMA)-9:1:0.05/DNA and HSPC:DOTAP:P(SMA-co-OEGMA)-9:1:0.1/DNA lipoplexes. The lipoplexes formed by pure liposomes and DNA were utilized as reference in order to investigate whether the presence of the copolymer in different molar ratios affect the formation and properties of the chimeric lipoplexes. Both types of lipoplexes display monomodal and relatively narrow size distributions in the whole N/P range, indicating the homogeneity of the formed lipoplex nanostructures. The derived size distributions of the above-mentioned types of lipoplexes at the examined N/P ratios are included in Figures S4–S6, respectively. In general, the lipoplexes in Figure 4 formed either by pure or chimeric liposomes and DNA exhibit a similar behavior as far as the variations of the R_h_ (Figure 4a–c), the scattered intensity (Figure 4d–f) and the zeta-potential (Figure 4g–i) are concerned. In most cases, the size of the lipoplexes (R_h_), as well as the scattered light intensity are gradually decreased as the N/P ratio increases from 0.25 to 8. Furthermore, the sizes of pure and chimeric lipoplexes at the whole range of the studied N/P ratios are larger compared to their parent liposomes, indicating the successful formation of lipoplexes in a wide range of N/P ratios. The decrease of the R_h_, accompanied by the parallel decrease of the scattered light intensity upon increasing N/P ratio, denotes the formation of lipoplexes of smaller size and lower molar mass. Particularly, in the case of the chimeric lipoplexes, at low N/P ratios (N/P < 1), the excess of DNA phosphate groups compared to the positive charges of DOTAP, favors the formation of larger nanostructures of high molar mass. In contrast, going to higher N/P ratios (N/P > 1), the number of available positive charges increase, thus the electrostatic interactions between DOTAP and DNA are more intense, resulting in the formation of more compact lipoplex nanostructures. Furthermore, the chimeric HSPC:DOTAP:P(SMA-co-OEGMA)-9:1:0.1/DNA lipoplexes presented smaller R_h_ and lower values of the scattered intensity in the whole range of N/P ratios, in comparison with the HSPC:DOTAP:P(SMA-co-OEGMA)-9:1:0.05/DNA lipoplexes. In this case, it seems that the higher content of the lipoplexes in the amphiphilic copolymer prompted the formation of smaller and compact nanostructures.

The surface charge of the lipoplexes was estimated by electrophoretic light scattering, to elucidate the successful formation of the lipoplexes. As it can be seen in Figure 4g–i, the lipoplexes display similar behavior as the N/P ratio increases from 0.25 to 8. At the N/P ratios of 0.25 and 0.5 both types of lipoplexes present negative zeta-potential values, due to the prevailing negative charges of the phosphate groups. In contrast, upon increasing the N/P ratio above the neutralization point (N/P > 1), the transition of the surface charge from negative to positive values signifies that the majority of the available positive charges of liposomes have efficiently interacted with DNA. Furthermore, it is evident that the chimeric lipoplexes exhibit less positive zeta-potential values in comparison to those of the pure lipoplexes and even to those of their parent liposomes, which is evidence of the effective shielding effect provided by the OEGMA chains of the copolymer.

**Figure 5:**
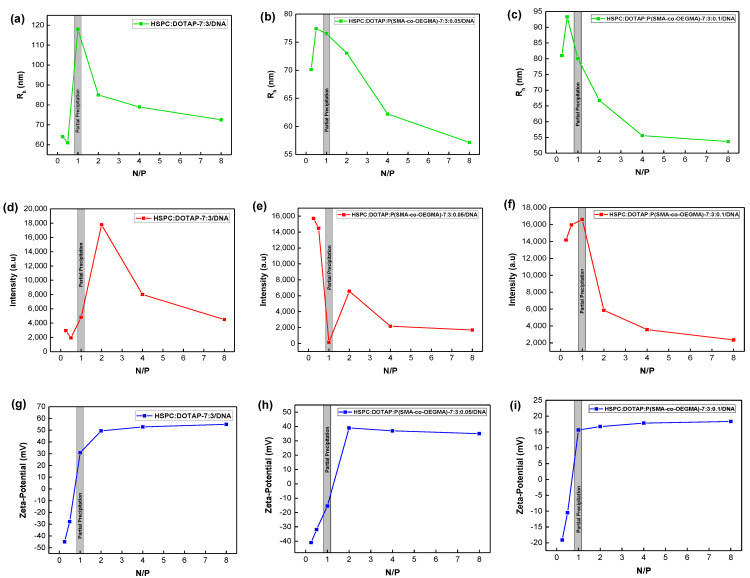
Variations of hydrodynamic radius (R_h_) with N/P ratio for (**a**) HSPC:DOTAP-7:3/DNA**,** (**b**) HSPC:DOTAP-7:3:0.05/DNA and (**c**) HSPC:DOTAP-7:3:0.1/DNA lipoplexes. Variations of scattered light intensity with N/P ratio for (**d**) HSPC:DOTAP-7:3/DNA, (**e**) HSPC:DOTAP-7:3:0.05/DNA, and (**f**) HSPC:DOTAP-7:3:0.1/DNA lipoplexes. Variations of zeta-potential with N/P ratio for (**g**) HSPC:DOTAP-7:3/DNA, (**h**) HSPC:DOTAP-7:3:0.05/DNA, and (**i**) HSPC:DOTAP-7:3:0.1/DNA lipoplexes.

Figure 5 depicts the obtained DLS and ELS results, concerning the size, intensity, and zeta-potential of the HSPC:DOTAP-7:3/DNA, HSPC:DOTAP:P(SMA-co-OEGMA)-7:3:0.05/DNA, and HSPC:DOTAP:P(SMA-co-OEGMA)-7:3:0.1/DNA lipoplexes, as a function of the N/P ratio. The complexation between the liposomes with the molar ratio of DOTAP prevailing resulted in the formation of lipoplex nanostructures with homogeneity in the whole N/P range, as their monomodal and narrow size distributions revealed (Figures S7–S9, respectively).

According to Figure 5, the variations in the size (R_h_) (Figure 5a–c), the scattered light intensity (Figure 5d–f) and the zeta-potential values (Figure 5g–i) of the pure and chimeric lipoplexes are generally following a pattern behavior similar to the one described in the case of the pure and chimeric lipoplexes composed of the lower molar ratio in DOTAP. Regarding the chimeric HSPC:DOTAP:P(SMA-co-OEGMA)-7:3:0.05/DNA and HSPC:DOTAP:P(SMA-co-OEGMA)-7:3:0.1/DNA lipoplexes, the decrease of the R_h_ along with the parallel decrease of the scattered light intensity, as the N/P ratio rises especially from 0.5 to 8, signals the assembly of lipoplexes with smaller size and lower molar mass. Furthermore, both HSPC:DOTAP:P(SMA-co-OEGMA)-7:3:0.05/DNA and HSPC:DOTAP:P(SMA-co-OEGMA)-7:3:0.1/DNA chimeric lipoplexes display similar values of the R_h_ and scattered light intensity at the majority of the N/P ratios. Moreover, their size, depending on the N/P ratio, is larger compared to the neat chimeric liposomes, indicating the efficacious formation of lipoplexes. The HSPC:DOTAP-7:3/DNA lipoplexes present higher values of scattered light intensity in comparison to the HSPC:DOTAP:P(SMA-co-OEGMA)-7:3:0.05/DNA, HSPC:DOTAP:P(SMA-co-OEGMA)-7:3:0.1/DNA chimeric lipoplexes, and also to the pure HSPC:DOTAP-9:1/DNA lipoplexes, implying that the increased molar ratio of DOTAP leads to the formation of nanostructures with higher molar mass.

The surface charge of the pure HSPC:DOTAP-7:3/DNA and chimeric HSPC:DOTAP:P(SMA-co-OEGMA)-7:3:0.05/DNA, HSPC:DOTAP:P(SMA-co-OEGMA)-7:3:0.1/DNA lipoplexes, similarly to the lipoplexes comprised of the same molar ratio of DOTAP, display negative values of zeta-potential at N/P ratios with an excess of DNA phosphate groups (N/P < 1) and acquires positive values upon shifting to N/P ratios (N/P < 1) with a higher number of available DOTAP positive charges. Hence, bearing in mind the zeta-potential values of HSPC:DOTAP-7:3/DNA lipoplexes and observing the zeta-potential values of the chimeric lipoplexes in Figure 5h,i, it is discerned that even the small addition of the copolymer (molar ratio 0.05) drastically reduced the highly positive surface charge compared to the pure lipoplexes. Furthermore, this reduction is more distinct in the case of the chimeric HSPC:DOTAP:P(SMA-co-OEGMA)-7:3:0.1/DNA system, which is comprised of higher molar ratio of the copolymer and thus greater content of the OEGMA segments. Undoubtedly, the presence of the OEGMA moieties efficiently decreased the surface charge of the lipoplexes by the shielding of the positive charges. Furthermore, it is worth noticing that although the HSPC:DOTAP:P(SMA-co-OEGMA)-7:3:0.1/DNA lipoplexes have larger positive charge due to the higher molar ratio of DOTAP (molar ratio 3), the presence of the copolymer in the HSPC:DOTAP:P(SMA-co-OEGMA)-7:3:0.1/DNA lipoplexes managed to tremendously reduce the surface charge by reaching values similar to those of HSPC:DOTAP:P(SMA-co-OEGMA)-9:1:0.1/DNA lipoplexes (Figure 5i), which are comprised of a lower molar ratio of DOTAP (molar ratio 1) and thus present fewer positive charges. This finding reveals the efficacious contribution of the copolymer in the fabrication of liposomes with strong binding affinity to DNA and then the formation of lipoplexes with controllable surface charge. Hence, the decrease of the positive charges without affecting drastically the binding affinity and transfection efficiency is an issue of great importance in the design of effective and safe non-viral gene delivery vectors. Therefore, the choice of the OEGMA segment as an alternate to PEG was successful, due to the efficient decrease of the highly positive surface charge without affecting the binding affinity of the chimeric liposomes to the DNA, as was revealed by the fluorescence spectroscopy and the total displacement of the EtBr from the DNA double helix. This finding is also of great importance due to the fact that usually the shielding of the positive charges leads to weakening of the binding affinity of the nanocarrier to the nucleic acid.

**Figure 6:**
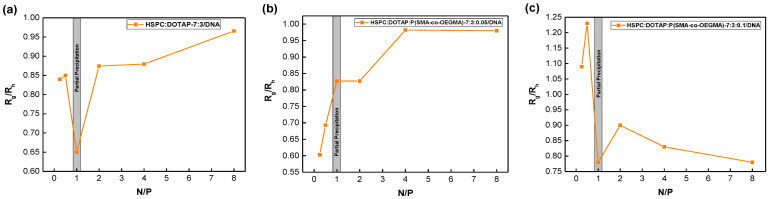
Variations of R_g_/R_h_ ratio as a function of N/P ratio for (**a**) HSPC:DOTAP-7:3/DNA, (**b**) HSPC:DOTAP-7:3:0.05/DNA, and (**c**) HSPC:DOTAP-7:3:0.1/DNA lipoplexes.

Additionally, static light scattering measurements were performed aiming to gain a first view on the morphology of the formed lipoplexes at the different N/P ratios, by determining the R_g_/R_h_ ratio. All the examined lipoplexes displayed similar behavior in the variations of the R_g_/R_h_ upon increasing N/P ratio. Representative plots presenting the variations of the R_g_/R_h_ ratio as a function of the N/P ratio are given in Figure 6a–c for the HSPC:DOTAP-7:3/DNA, HSPC:DOTAP-7:3:0.05/DNA, and HSPC:DOTAP-7:3:0.1/DNA lipoplexes, respectively. In the case of the pure HSPC:DOTAP-7:3/DNA (Figure 6a), as the N/P ratio rises from 0.25 to 8, accordingly the values of the R_g_/R_h_ ratio increase from approximately 0.84 to 0.95. These values indicate the formation of lipoplexes with mostly vesicular morphology at these N/P ratios. Similarly to HSPC:DOTAP-7:3/DNA lipoplexes, the R_g_/R_h_ ratio of the chimeric HSPC:DOTAP-7:3:0.05/DNA lipoplexes (Figure 6b) also increases by going to higher N/P ratios The obtained values suggest the formation of more compact spherical nanostructures at the N/P ratios with excess of DNA phosphate groups and the transition to more loose vesicular nanostructures upon increasing the N/P ratio. On the other hand, the HSPC:DOTAP:P(SMA-co-OEGMA)-7:3:0.1/DNA lipoplexes (Figure 6c) exhibit a decrease of their R_g_/R_h_ values as the N/P ratio increases, particularly from 0.5 to 8. In this N/P ratio range, the R_g_/R_h_ ratio obtained values ranging from ca. 1.23 at N/P = 0.5 to 0.78 at N/P = 8. These values show the formation of lipoplexes with loose conformation [63] at low N/P ratios, while at higher N/P ratios the lipoplexes assemble into more compact nanostructures with overall globular morphology.

Summarizing, light scattering findings revealed the efficient interaction of all the examined pure and chimeric liposomes with the DNA and therefore the successful formation of lipoplexes, whose physicochemical characteristics, colloidal stability, and morphology strongly depend on and are affected by the N/P ratio and especially on the molar ratio of the cationic lipid and the amphiphilic copolymer. Hence, the appropriate design focusing on the delicate balance of these parameters which affect the formation and the performance of the lipoplexes can provide the perspectives to the HSPC:DOTAP: P(SMA-co-OEGMA) liposomal formulations to be utilized as non-viral vectors for DNA delivery as well as other types of nucleic acids.

### 3.5. Influence of Ionic Strength on the Stability of the Lipoplexes

The ionic strength of the biological fluids affects the physicochemical characteristics of the nanocarriers by causing changes in their size, surface charge, and therefore to their colloidal stability [16]. Hence, the influence of the physiological conditions of the biological fluids on the nanocarriers is significant and it should be considered during the fabrication of effective gene nanocarriers.

The behavior of the lipoplexes in the presence of salt was examined by adding NaCl of 1 M and gradually increasing the salt concentration from 0 M to 0.5 M. DLS was employed to monitor changes on the size and scattered light intensity of the lipoplexes.

The tolerance of the pure and chimeric lipoplexes on increasing ionic strength was selected to be examined at N/P ratios above the precipitation region (N/P > 1) with optimal colloidal stability for at least one week. Furthermore, nanocarriers with a small excess of positive charges are more suitable for nucleic acid delivery due to the fact that the free positive charges facilitate the intracellular uptake by interacting with the negative charges of the cellular membrane [19,20]. In this regard, the influence of the ionic strength on the lipoplexes was investigated at the ratio N/P = 4.

**Figure 7:**
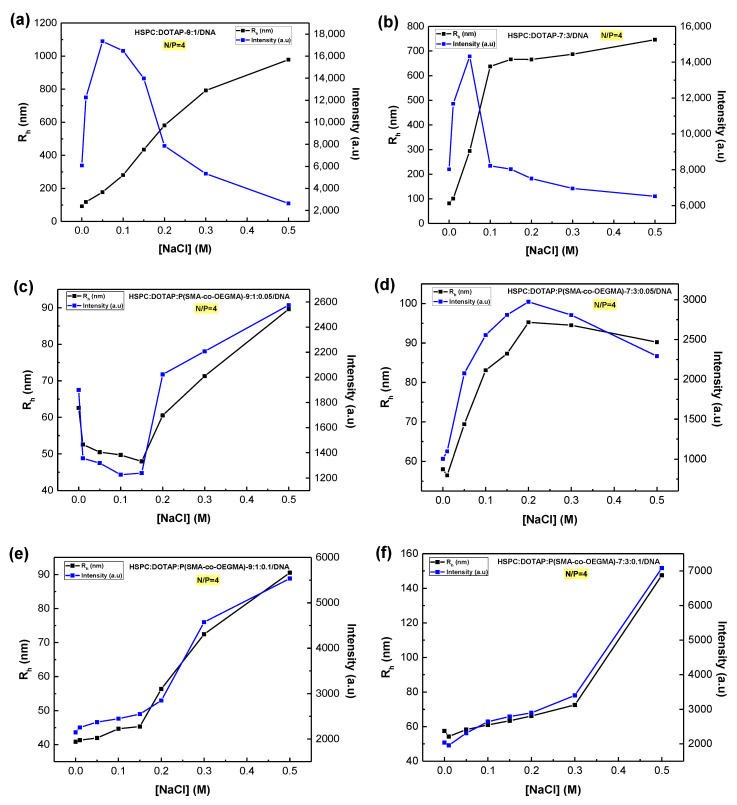
Hydrodynamic radius (R_h_) and scattered light intensity as a function of ionic strength for (**a**) HSPC:DOTAP-9:1/DNA, (**b**) HSPC:DOTAP-7:3/DNA, (**c**) HSPC:DOTAP-9:1:0.05/DNA, (**d**) HSPC:DOTAP-7:3:0.05/DNA, (**e**) HSPC:DOTAP-9:1:0.1/DNA, and (**f**) HSPC:DOTAP-9:1:0.05/DNA lipoplexes at the ratio N/P = 4.

The variations of the hydrodynamic radius (R_h_) and the scattered light intensity of the pure and chimeric lipoplexes as a function of the ionic strength, at N/P = 4, are depicted on Figure 7. In the case of the pure HSPC:DOTAP-9:1/DNA lipoplexes (Figure 7a), R_h_ gradually increases reaching almost 1000 nm at the final salt concentration of 0.5 M. On the other hand, a parallel increase of the scattered light intensity is observed up to the salt concentration of 0.05 M and thereafter declines until the concentration of 0.5 M. The parallel increase of the R_h_ and the scattered intensity denote the growth of the lipoplexes in size and mass, while the decrease of the intensity from the concentration of 0.05 M till 0.5 M indicates the reduction in their mass. Particularly, the simultaneous increase in size and decrease in mass as the salt concentration rises, signifies the disintegration and destabilization of the lipoplexes in the presence of higher salt concentrations.

Similar tendency in the variations of the R_h_ and the scattered intensity was exhibited by the HSPC:DOTAP-7:3/DNA lipoplexes (Figure 7b). Specifically, R_h_ along with the scattered intensity simultaneously increases until 0.05 M, then the intensity decreases and the R_h_ rises, reaching ca. 750 nm at the concentration of 0.5 M NaCl. The addition of salt induces charge screening effects and leads to the weaking of the affinity between the cationic liposomes and the DNA. In this way, the solubility of the lipoplexes is enhanced, resulting in their swelling due to the insertion of water molecules within their structures. Hence, the addition of salt caused the rapid increase of the R_h_ of both pure lipoplexes and resulted in large sizes and high intensities which is evidence of lack of their colloidal stability, even at low NaCl concentrations.

As far as the chimeric lipoplexes are concerned, it can be observed in Figure 7c that the HSPC:DOTAP:P(SMA-co-OEGMA)-9:1:0.05/DNA lipoplexes display a slight decrease of the R_h_, accompanied by the decrease of the intensity until 0.15 M. Nevertheless, these variations of R_h_ and intensity are small, thus the lipoplexes remain essentially stable until 0.15 M NaCl. However, the parallel increase of R_h_ and I after 0.15 M until 0.5 M evidences the increase of lipoplexes in size and mass, but not their decomposition. In contrast, HSPC:DOTAP:P(SMA-co-OEGMA)-7:3:0.05/DNA lipoplexes (Figure 7d) present simultaneous increase of the R_h_ and scattered light intensity even from the first addition of salt, demonstrating that the structure of the lipoplexes may became looser and a tendency to aggregation. Similar behavior pattern is also observed in the case of HSPC:DOTAP:P(SMA-co-OEGMA)-9:1:0.1/DNA (Figure 7e) and HSPC:DOTAP:P(SMA-co-OEGMA)-7:3:0.1/DNA (Figure 7f) chimeric lipoplexes. The stability of the lipoplexes in the presence of salt is more evident in the case of the HSPC:DOTAP:P(SMA-co-OEGMA)-7:3:0.1/DNA (Figure 7f). The R_h_ and intensity remain rather constant until 0.2 M and thereafter increase sharply from 0.3 M to 0.5 M, indicating farther aggregation and destabilization.

Overall, the chimeric lipoplexes were found to partially tolerate an increase in ionic strength, and to retain their complexation ability and colloidal stability under physiological salinity (equivalent to ca. 0.15 M NaCl). In contrast, even at low salt concentration, the pure lipoplexes exhibited large R_h_ and intensity values. Hence, the increase of ionic strength strongly affected the stability of the pure lipoplexes, resulting in their swelling and thereafter their decomposition.

In conclusion, the tolerance of the chimeric lipoplexes under the influence of the ionic strength is attributed to the presence of the copolymer, which noticeably contributed to lipoplexes stability. Particularly, the hydrophobic interactions provided by the long alkyl chains of the SMA segments, as well as the stealth/stabilization effect of the OEGMA segments, enhanced the colloidal stability of the chimeric lipoplexes and prevented their total collapse at higher salt concentrations. Moreover, the chimeric lipoplexes with the higher copolymer molar ratio (0.1) displayed smaller variations on their size and mass and thus exhibited a better stability profile under physiological salinity, compared to the chimeric lipoplexes with the lower copolymer molar ratio (0.05). Consequently, these findings evidence the efficient contribution of the amphiphilic copolymer in the lipoplex formulations.

### 3.6. Morphological Characterization of Chimeric Liposomes and Lipoplexes via Cryo-TEM

**Figure 8:**
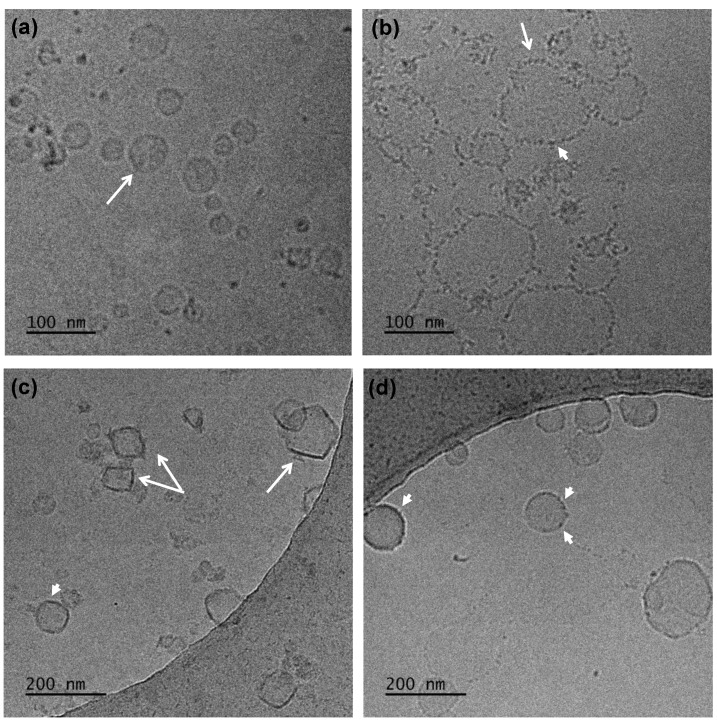
Representative Cryo-TEM images of (**a**) HSPC:DOTAP:P(SMA-co-OEGMA)-9:1:0.1 chimeric liposomes, (**b**) HSPC:DOTAP:P(SMA-co-OEGMA)-9:1:0.1/DNA chimeric lipoplexes at ratio N/P = 4, (**c**) HSPC:DOTAP:P(SMA-co-OEGMA)-7:3:0.1 chimeric liposomes, and (**d**) HSPC:DOTAP:P(SMA-co-OEGMA)-7:3:0.1/DNA chimeric lipoplexes at ratio N/P = 4.

The morphology and the internal structure of the prepared chimeric liposomes as well as the chimeric lipoplexes were evaluated by Cryo-TEM. Representative Cryo-TEM images of the obtained nanostructures are provided in Figure 8. In the case of HSPC:DOTAP:P(SMA-co-OEGMA)-9:1:0.1 liposomes, the cryo-TEM micrograph (Figure 8a) evidenced the formation of vesicular structures of sizes varying from 20–290 nm with average size 58 nm (measured for 100 objects). Furthermore, the liposomes present unilamellar structure and circular shape (long white arrow in Figure 8a). The lipid membrane can be easily distinguished due to the different contrast between the periphery and the cavity of the liposomes. Moreover, the thickness of the membrane is 5 to 8 nm. It should be noted that the HSPC:DOTAP:P(SMA-co-OEGMA)-9:1:0.05 liposomes (data not shown) displayed similar morphology and structure, which indicates that the copolymer did not dramatically affect the morphological characteristics of the chimeric liposomes. Figure 8b depicts the lipoplexes formed by the electrostatic interaction of the HSPC:DOTAP:P(SMA-co-OEGMA)-9:1:0.1 liposomes with the DNA of 113 bp, at the N/P = 4 ratio. The formation of structures with morphology different than the precursor chimeric liposomes is observed. The sizes of lipoplexes range from 20 to 200 nm, with an average size of 73 nm (measured for 100 objects) and the thickness of their membrane is 5–7 nm. Furthermore, the outer surface of the lipoplexes presents a dot-like halo shape (long white arrow in Figure 8b) with several dark spots on the periphery of the membrane (short white arrow in Figure 8b). These dark spots of size 5–10 nm can be probably assigned to domains where DNA molecules are complexed, which may be visible due to the high contrast of the phosphorus atoms of DNA. Moreover, these spots are not detected in the precursor liposomes. This observation indicates the presence of DNA and thus the successful formation of lipoplexes. HSPC:DOTAP:P(SMA-co-OEGMA)-9:1:0.05/DNA lipoplexes presented the same dot-like membrane shape with the dark spots, as the one observed for the HSPC:DOTAP:P(SMA-co-OEGMA)-9:1:0.1/DNA lipoplexes (Figure 8b).

The chimeric liposomes with higher lipid molar ratio in DOTAP, namely the HSPC:DOTAP:P(SMA-co-OEGMA)-7:3:0.1 (Figure 8c), exhibited vesicular and unilamellar morphology with different shapes. Particularly, the co-existence of nanostructures with spherical (short white arrows in Figure 8c) and polygon-like (long white arrows in Figure 8c) shape is detected, in comparison to the HSPC:DOTAP:P(SMA-co-OEGMA)-9:1:0.1 liposomes which formed only spherical structures. The sizes of the liposomes were between 20 and 145 nm and average size of 66 nm (measured for 100 objects), while the thickness of the membrane was 5–8 nm. Furthermore, the HSPC:DOTAP:P(SMA-co-OEGMA)-7:3:0.05 liposomes presented the same spherical and polygon-like shape with the HSPC:DOTAP:P(SMA-co-OEGMA)-7:3:0.1 liposomes. It is also evident that the molar ratio of the copolymer did not affect the obtained liposomes morphology, in contrast to the molar ratio of DOTAP, which its increase caused differences in the liposomes shape. The HSPC:DOTAP:P(SMA-co-OEGMA)-7:3:0.1/DNA chimeric lipoplexes formed at the ratio N/P = 4 are presented in Figure 8d. The formation of mainly vesicular structures with thick lipid membranes is observed. Moreover, the lipoplexes exhibit mainly spherical shape and larger sizes of 30–265 nm (average size: 122 nm, measured for 100 objects), compared to their precursor liposomes. The thickness of the membrane for the lipoplexes ranges from 5–8 nm. Similar to the HSPC:DOTAP:P(SMA-co-OEGMA)-9:1:0.1/DNA lipoplexes, dark spots on the periphery of the lipid membrane of HSPC:DOTAP:P(SMA-co-OEGMA)-7:3:0.1/DNA lipoplexes are also observed. Moreover, these dark spots with sizes between 5–10 nm are more intense in some sites of the membrane surface (short white arrows). The high density of these spots maybe indicates that larger amount of DNA molecules is located/complexed on these sites.

Consequently, the morphological and structural characteristics of chimeric liposomes and lipoplexes are strongly dependent on the lipid ratio. Additionally, it should be noted that the obtained vesicular morphologies for most of the studied nanosystems by Cryo-TEM, are in accordance with the determined R_g_/R_h_ ratios by light scattering. Hence, the lipoplexes, due to their small size and vesicular morphology, are expected to facilitate the delivery of DNA.

## 4. Conclusions

In this study, a novel hybrid-chimeric liposomal nanoplatform composed of the HSPC, DOTAP lipids, and the P(SMA-co-OEGMA) copolymer was developed and was further investigated as a potential nanocarrier for nucleic acid delivery. The implementation of RAFT polymerization technique resulted in the fabrication of a well-defined amphiphilic random copolymer P(SMA-co-OEGMA) with desirable and controllable molecular characteristics. The hybrid/chimeric liposomal nanosystems were successfully prepared by thin film method in several molar ratios of the copolymer and lipid components and compared to pure liposomes composed of HSPC and DOTAP lipids. Light scattering techniques revealed that all the prepared liposomes presented well-defined physicochemical characteristics. The chimeric liposomes as well as the pure liposomes were successfully explored for their ability to electrostatically interact with a model DNA of 113 bp, by forming lipoplexes. UV–Vis and especially fluorescence spectroscopy proved the strong binding affinity of the cationic liposomes to DNA and the formation of lipoplexes, by the total displacement of the intercalated EtBr from the double helix of DNA, in the latter studies. DLS, ELS, and SLS measurements proved that nanosized pure/chimeric lipoplexes were formed in a wide range of N/P ratios. The findings proved that the utilization of OEGMA is a valuable alternative to PEG and can be utilized as an approach to reduce possible cytotoxicity effects due to the incorporated cationic lipids. The stability of the lipoplexes under the influence of increasing solution ionic strength, as a preliminary simulation of the physiological conditions of the biological fluids, was investigated by dynamic light scattering. The chimeric lipoplexes were found to tolerate the increase of the ionic strength and to retain their colloidal stability and complexation state under physiological salinity. In contrast, the pure lipoplexes presented large sizes, indicating their disintegration and destabilization in the presence of salt, even at low salt concentrations. Consequently, the amphiphilic copolymer efficiently contributed and enhanced the stabilization of the chimeric lipoplexes in the presence of salt. Cryo-TEM images revealed the formation of vesicular nanostructures, with their shape depending on the lipid ratio. The presence of DNA molecules on the periphery of the lipid membrane evidenced the successful formation of lipoplexes, with their shape also depending on the lipid ratio.

In conclusion, the fabrication of a novel chimeric liposomal nanosystem in different molar ratios of its components and the interaction with DNA resulted in colloidally stable chimeric lipid-copolymer lipoplexes with well-defined physicochemical characteristics. Their size, surface charge, and morphology are strongly depending on the N/P ratio and particularly on the molar ratio of each component. The contribution of the copolymer on the well-defined characteristics was instrumental. The significant progress in polymer chemistry has opened the access to the design and fabrication of advanced polymeric materials with well-defined characteristics and desirable features. With the present study, we highlight the importance of mixing different materials. i.e., polymers and lipids. The synergistic effect of different in nature materials paves the road on the exploration and development of multifunctional nanocarriers as a new promising therapeutic approach in gene therapy.

## Figures and Tables

**Table 1:** Physicochemical characteristics of the developed nanosystems, as determined by light scattering techniques.

Sample	Molar Ratio	R_h_ (nm) ^a^	PDI ^a^	R_g_/R_h_^b^	Z_p_ (mV) ^c^
HSPC:DOTAP	9:1	71	0.21	0.80	+44
HSPC:DOTAP:P(SMA-co-OEGMA)	9:1:0.05	46	0.45	1.10	+25
HSPC:DOTAP:P(SMA-co-OEGMA)	9:1:0.1	33	0.43	1.11	+22
HSPC:DOTAP	7:3	70	0.22	0.96	+56
HSPC:DOTAP:P(SMA-co-OEGMA)	7:3:0.05	55	0.29	1.08	+51
HSPC:DOTAP:P(SMA-co-OEGMA)	7:3:0.1	57	0.36	1.02	+45

^a^ Determined by DLS and CONTIN analysis at 90°, ^b^ Determined by SLS, ^c^ Determined by ELS.

## Data Availability

The data presented in this study are available upon request.

## Supplementary Materials

The following supporting information can be downloaded at: https://www.mdpi.com/article/10.3390/polym14224901/s1, Figure S1: UV–vis absorption spectra of (a) HSPC:DOTAP-9:1/DNA, (b) HSPC:DOTAP-7:3/DNA, (c) HSPC:DOTAP:P(SMA-co-OEGMA)-9:1:0.05/DNA, (d) HSPC:DOTAP:P(SMA-co-OEGMA)-7:3:0.05/DNA, (e) HSPC:DOTAP:P(SMA-co-OEGMA)-9:1:0.1/DNA, (f) HSPC:DOTAP:P(SMA-co-OEGMA)-7:3:0.1/DNA lipoplexes, for N/P ratios ranging from 0.25 to 8, Figure S2: UV–Vis spectra of pure HSPC:DOTAP and chimeric HSPC:DOTAP:P(SMA-co-OEGMA) liposomes in aqueous media, Figure S3: Fluorescence spectra of the intercalated EtBr for (a) HSPC:DOTAP-9:1/DNA, (b) HSPC:DOTAP-7:3/DNA, (c) HSPC:DOTAP:P(SMA-co-OEGMA)-9:1:0.05/DNA, (d) HSPC:DOTAP:P(SMA-co-OEGMA)-7:3:0.05/DNA, (e) HSPC:DOTAP:P(SMA-co-OEGMA)-9:1:0.1/DNA, and (f) HSPC:DOTAP:P(SMA-co-OEGMA)-7:3:0.1/DNA lipoplexes at different N/P ratios, Figure S4. Size distributions from CONTIN analysis of HSPC:DOTAP-9:1/DNA lipoplexes, at ratios (a) N/P = 0.25, (b) N/P = 0.5, (c) N/P = 1 (partial precipitation takes place at N/P = 1, data shown are from supernatant solution), (d) N/P = 2, (e) N/P = 4, and (f) N/P = 8, Figure S5. Size distributions from CONTIN analysis of HSPC:DOTAP-9:1:0.05/DNA chimeric lipoplexes, at ratios (a) N/P = 0.25, (b) N/P = 0.5, (c) N/P = 1 (partial precipitation takes place at N/P = 1, data shown are from supernatant solution), (d) N/P = 2, (e) N/P = 4, and (f) N/P = 8, Figure S6. Size distributions from CONTIN analysis of HSPC:DOTAP-9:1:0.1/DNA chimeric lipoplexes, at ratios (a) N/P = 0.25, (b) N/P = 0.5, (c) N/P = 1 (partial precipitation takes place at N/P = 1, data shown are from supernatant solution), (d) N/P = 2, (e) N/P = 4 and (f) N/P = 8, Figure S7. Size distributions from CONTIN analysis of HSPC:DOTAP-7:3/DNA lipoplexes, at ratios (a) N/P = 0.25, (b) N/P = 0.5, (c) N/P = 1 (partial precipitation takes place at N/P = 1, data shown are from supernatant solution), (d) N/P = 2, (e) N/P = 4, and (f) N/P = 8, Figure S8. Size distributions from CONTIN analysis of HSPC:DOTAP-7:3:0.05/DNA chimeric lipoplexes, at ratios (a) N/P = 0.25, (b) N/P = 0.5, (c) N/P = 1 (partial precipitation takes place at N/P = 1, data shown are from supernatant solution), (d) N/P = 2, (e) N/P = 4, and (f) N/P = 8, Figure S9. Size distributions from CONTIN analysis of HSPC:DOTAP-7:3:0.1/DNA chimeric lipoplexes, at ratios (a) N/P = 0.25, (b) N/P = 0.5, (c) N/P = 1 (partial precipitation takes place at N/P = 1, data shown are from supernatant solution), (d) N/P = 2, (e) N/P = 4, and (f) N/P = 8.
