# Supplementary material for: Amphiphilic Copolymer-Lipid Chimeric Nanosystems as DNA Vectors
Source: Polymers (Basel). 2022 Nov 13;14(22):4901. doi: 10.3390/polym14224901 (PMC9699196; doi:10.3390/polym14224901)
Supplement: Supplementary file 1 [file polymers-14-04901-s001.zip › polymers-1999840-supplementary.pdf]

polymers
MDPI

Supplementary Materials

Amphiphilic Copolymer-Lipid Chimeric Nanosystems as DNA Vectors

Varvara Chrysostomou [1,2], Aleksander Foryś [3], Barbara Trzebicka [3], Costas Demetzos [1] and Stergios Pispas [2,*]

1 Section of Pharmaceutical Technology, Department of Pharmacy, School of Health Sciences, National and Kapodistrian University of Athens, Panepistimioupolis Zografou, 15771 Athens, Greece
2 Theoretical and Physical Chemistry Institute, National Hellenic Research Foundation, 48 Vassileos Constantinou Avenue, 11635 Athens, Greece
3 Centre of Polymer and Carbon Materials, Polish Academy of Sciences, 34 ul. M. Curie-Skłodowskiej, 41-819 Zabrze, Poland
* Correspondence: pispas@eie.gr; Tel.: +30-2107273824

1. Lipoplexes Absorption Spectra by UV-Vis Spectroscopy

UV-Vis spectroscopy was implemented in order to obtain qualitative information on the ability of the cationic liposomes to electrostatically interact with the DNA of 113bp and form lipoplexes. The absorption of DNA in the UV-Vis spectral range permits the identification of possible alterations in its conformation state, arising from the interaction with positively charged compounds. Particularly, DNA presents a broad band in the UV-Vis spectral region (200-350 nm), with a λ_{max} at 260 nm [1,2]. Upon interaction with the cationic liposomes and the formation of lipoplexes, the absorption intensity of the DNA at 260 nm decreases and a new peak appears in shorter wavelength, which is usually detected at approximately 225 nm [1,2].

The recorded UV-Vis spectra of the pure and chimeric liposomes complexing with the DNA and their formed lipoplexes at different N/P ratios ranging from 0.25-8 are presented in Figure S1. It is worthy of note that both types of liposomes do not display absorption peaks in the UV-Vis region (Figure S2). Furthermore, most of the prepared lipoplexes were partially precipitated at the neutralization point (N/P=1). Therefore, UV-Vis experiments were carried out by measuring the supernatant of the solutions. However, the UV-Vis spectra of the lipoplexes corresponding to the ratio N/P=1 should not be taken into consideration as they are not completely representative of the mixtures.

At first glance, none of the recorded UV-Vis spectra present a peak at shorter wavelength near the 225nm, which would implying the presence of bonded DNA and therefore the strong binding capacity of the cationic liposomes. However, there is insufficient information in the literature concerning the evaluation of the peak at ca. 225 nm. It is believed that this peak is related to configuration transitions of the DNA during the complexation process, since liposomes in this case do not exhibit any peaks in this particular spectral range.

However, some alterations in the absorbance of the peak at 260 nm which is assigned to the free/uncomplexed DNA can be observed and thus information regarding the interaction between the liposomes and the DNA can be extracted. More specifically, the absorption intensity of this peak varies according to the N/P ratio. The absorbance is expected to be sharper at N/P ratios with excess of phosphate groups due to the free DNA. However, at N/P ratios with excess of amine groups, the absorbance is decaying. This reduction in the absorbance of the DNA peak at N/P ratios above 1 maybe indicates that the available charges of the DNA phosphate groups interact efficiently with the amine groups of DOTAP and therefore large amount of free DNA is not detected at N/P ratios above 1. On the other hand, the absence of the peak at ca. 225 nm maybe implies the weak

electrostatic interaction and the low binding affinity of the liposomes to DNA. Another explanation of the non-detection of the peak at 225 nm is probably due to conformation changes of the arrangement of DNA in the lipoplexes. In other words, the complexed DNA molecules are possibly accommodated on the periphery of the outer surface of the liposomes and thus are detected like free DNA in the examined solutions, taking into account the fluidity/deformability of the liposomal membrane. More convincing evidence for lipoplex formation is given by fluorescence spectroscopy assays.

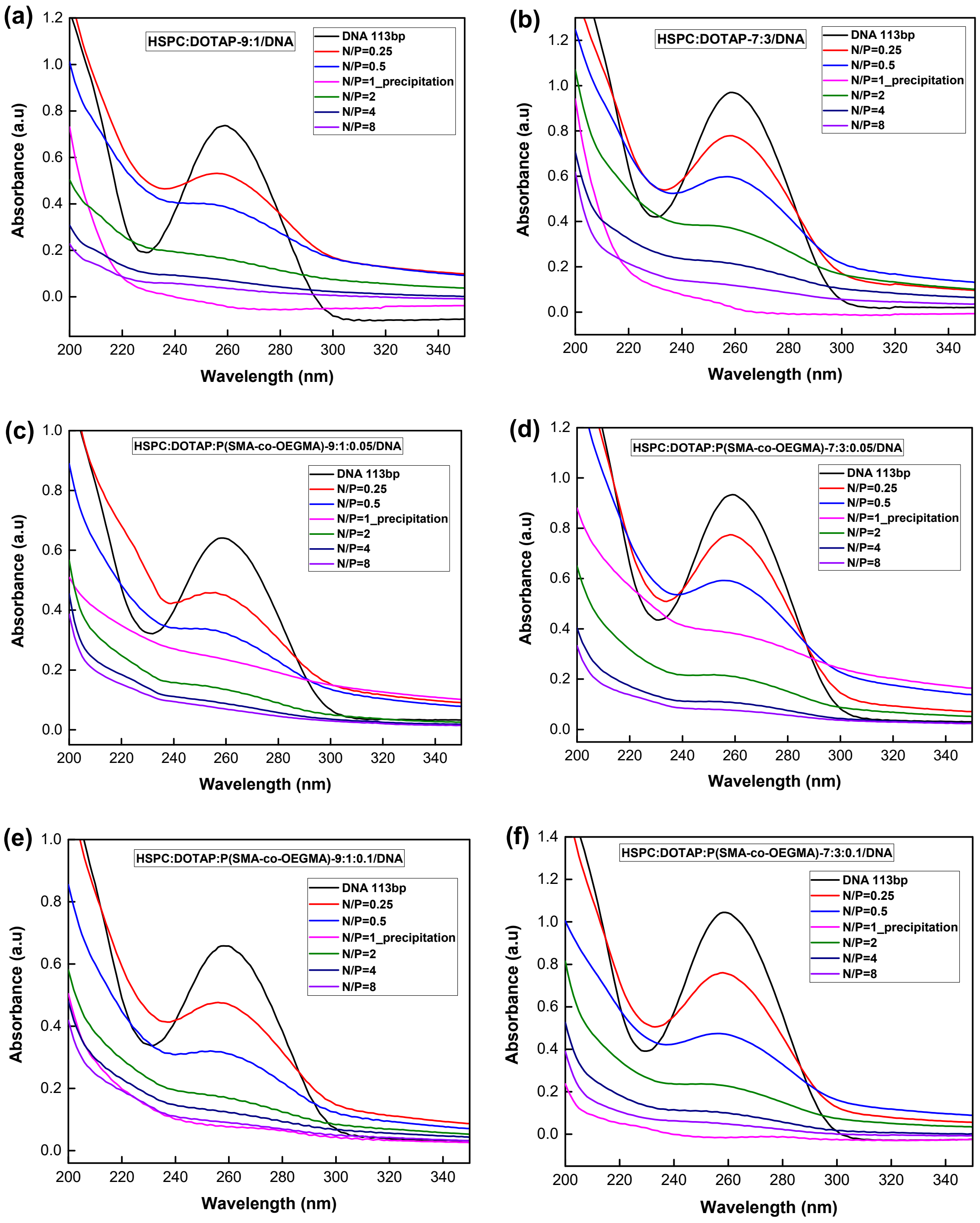

Figure S1. UV–vis absorption spectra of (**a**) HSPC:DOTAP-9:1/DNA, (**b**) HSPC:DOTAP-7:3/DNA, (**c**) HSPC:DOTAP:P(SMA-co-OEGMA)-9:1:0.05/DNA, (**d**) HSPC:DOTAP:P(SMA-co-OEGMA)-7:3:0.05/DNA, (**e**) HSPC:DOTAP:P(SMA-co-OEGMA)-9:1:0.1/DNA, (**f**) HSPC:DOTAP:P(SMA-co-OEGMA)-7:3:0.1/DNA lipoplexes, for N/P ratios ranging from 0.25 to 8.

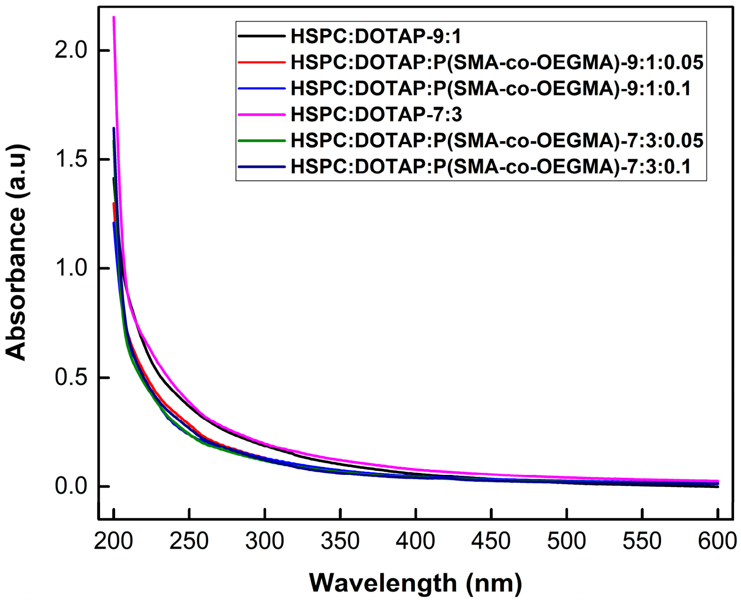

Figure S2. UV-Vis spectra of pure HSPC:DOTAP and chimeric HSPC:DOTAP:P(SMA-co-OEGMA) liposomes in aqueous media.

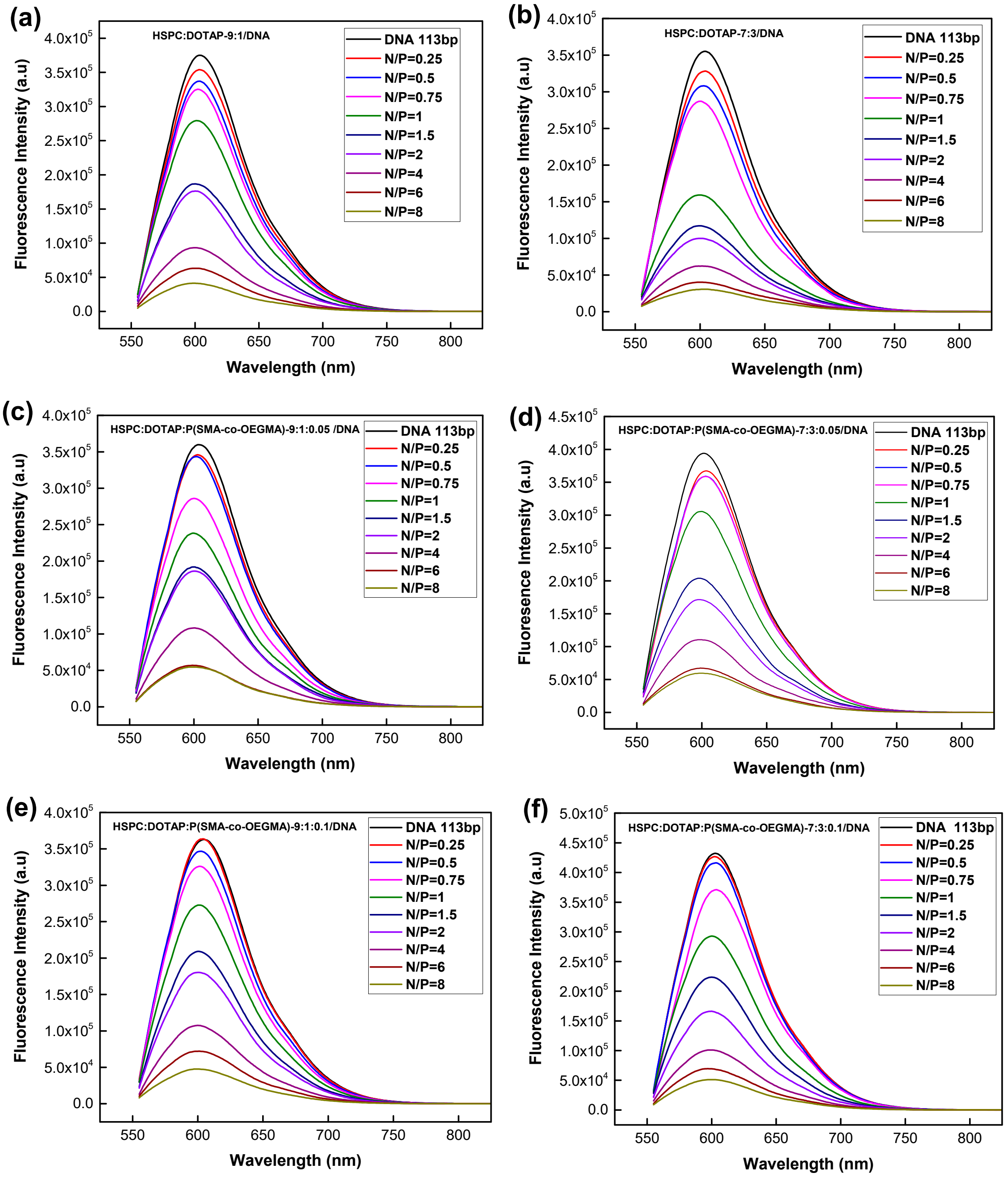

Figure S3. Fluorescence spectra of the intercalated EtBr for (**a**) HSPC:DOTAP-9:1/DNA, (**b**) HSPC:DOTAP-7:3/DNA, (**c**) HSPC:DOTAP:P(SMA-co-OEGMA)-9:1:0.05/DNA, (**d**) HSPC:DOTAP:P(SMA-co-OEGMA)-7:3:0.05/DNA, (**e**) HSPC:DOTAP:P(SMA-co-OEGMA)-9:1:0.1/DNA and (**f**) HSPC:DOTAP:P(SMA-co-OEGMA)-7:3:0.1/DNA lipoplexes at different N/P ratios.

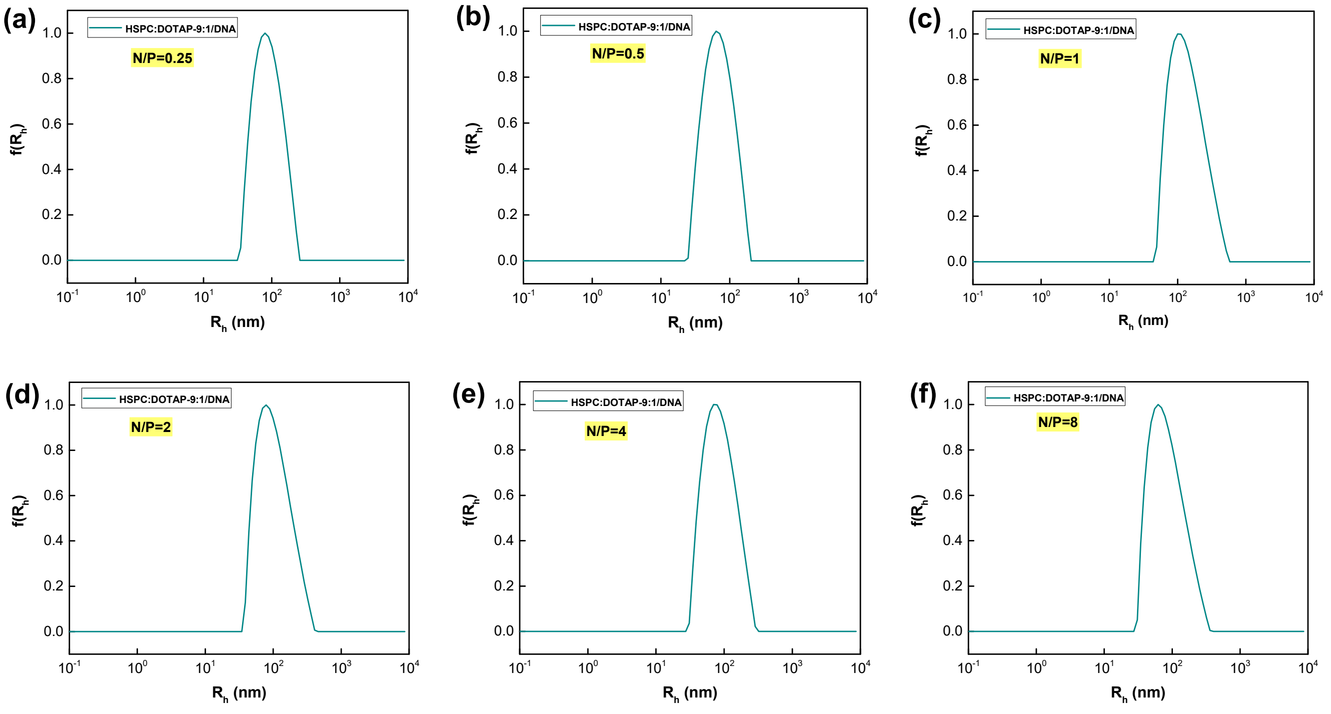

Figure S4. Size distributions from CONTIN analysis of HSPC:DOTAP-9:1/DNA lipoplexes, at ratios (**a**) N/P=0.25, (**b**) N/P=0.5, (**c**) N/P= 1 (partial precipitation takes place at N/P=1, data shown are from supernatant solution), (**d**) N/P=2, (**e**) N/P=4 and (**f**) N/P=8.

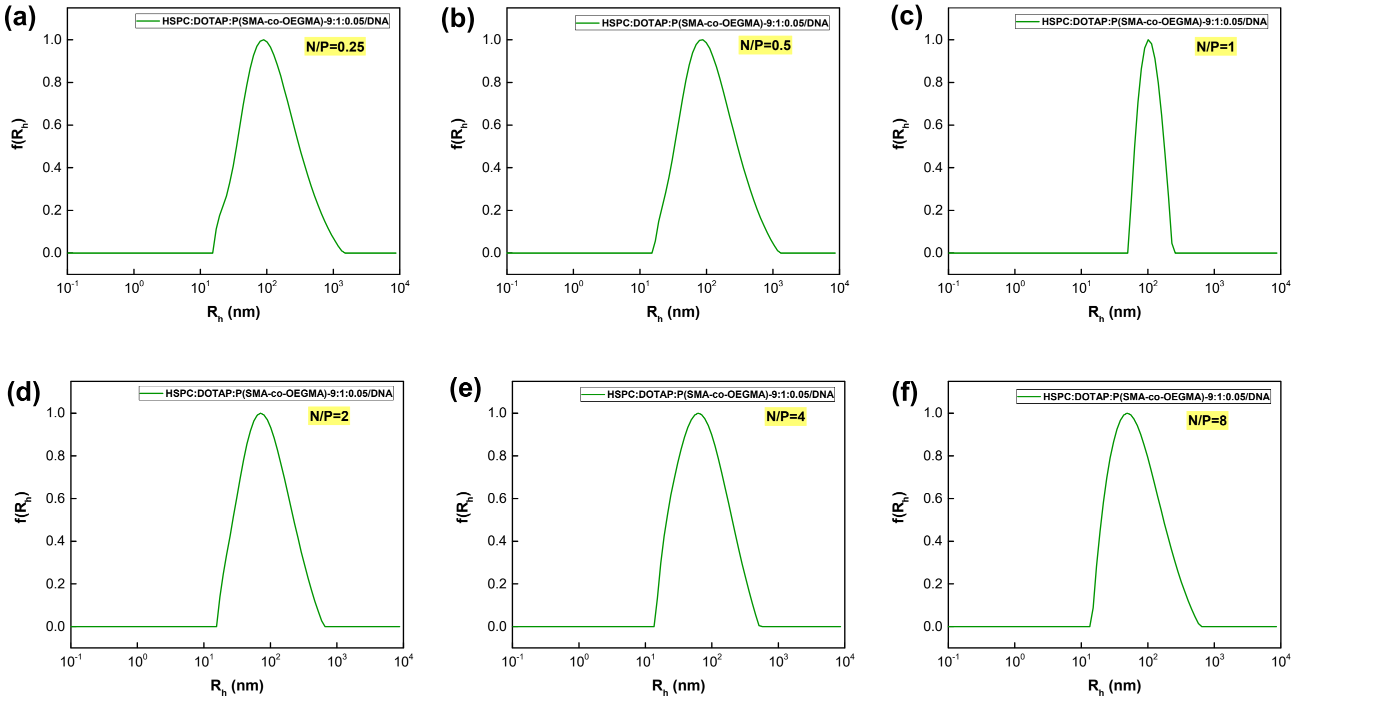

Figure S5. Size distributions from CONTIN analysis of HSPC:DOTAP-9:1:0.05/DNA chimeric lipoplexes, at ratios (**a**) N/P=0.25, (**b**) N/P=0.5, (**c**) N/P= 1 (partial precipitation takes place at N/P=1, data shown are from supernatant solution), (**d**) N/P=2, (**e**) N/P=4 and (**f**) N/P=8.

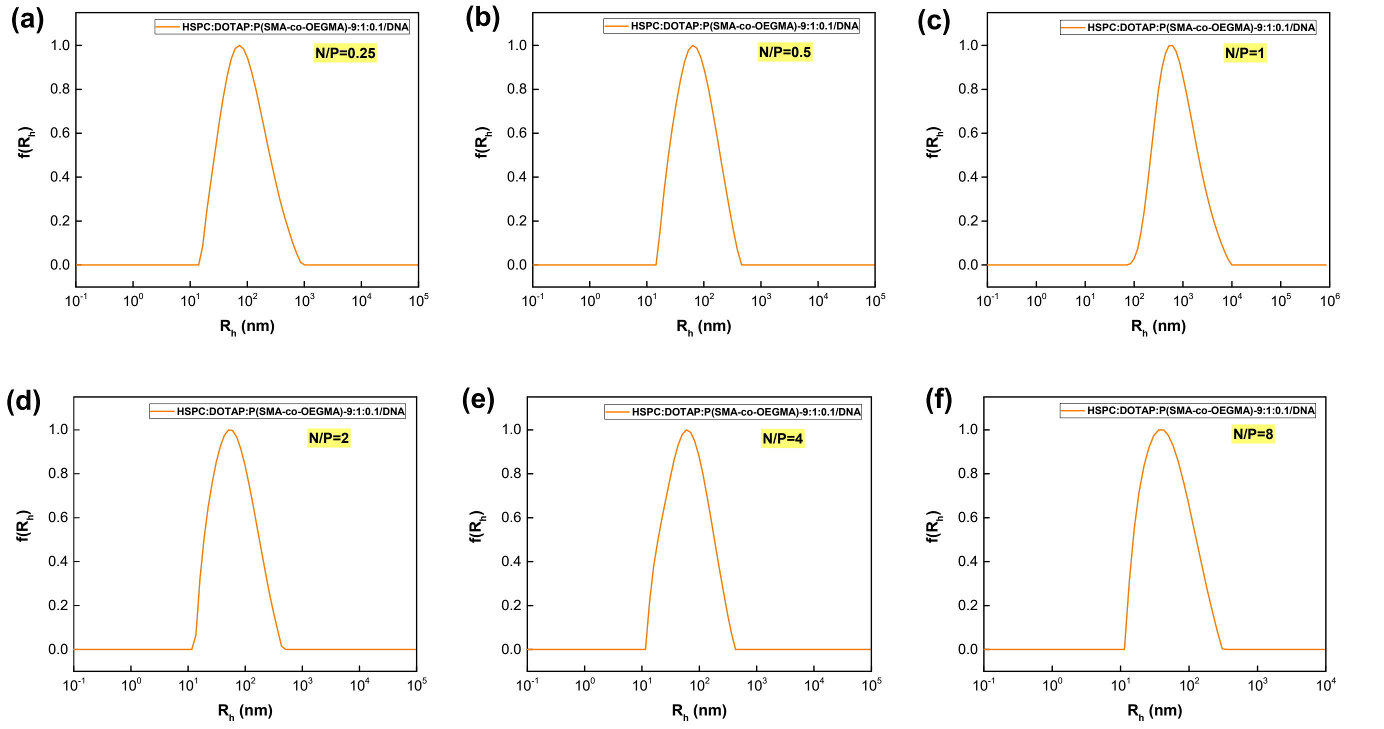

Figure S6. Size distributions from CONTIN analysis of HSPC:DOTAP-9:1:0.1/DNA chimeric lipoplexes, at ratios (**a**) N/P=0.25, (**b**) N/P=0.5, (**c**) N/P= 1 (partial precipitation takes place at N/P=1, data shown are from supernatant solution), (**d**) N/P=2, (**e**) N/P=4 and (**f**) N/P=8.

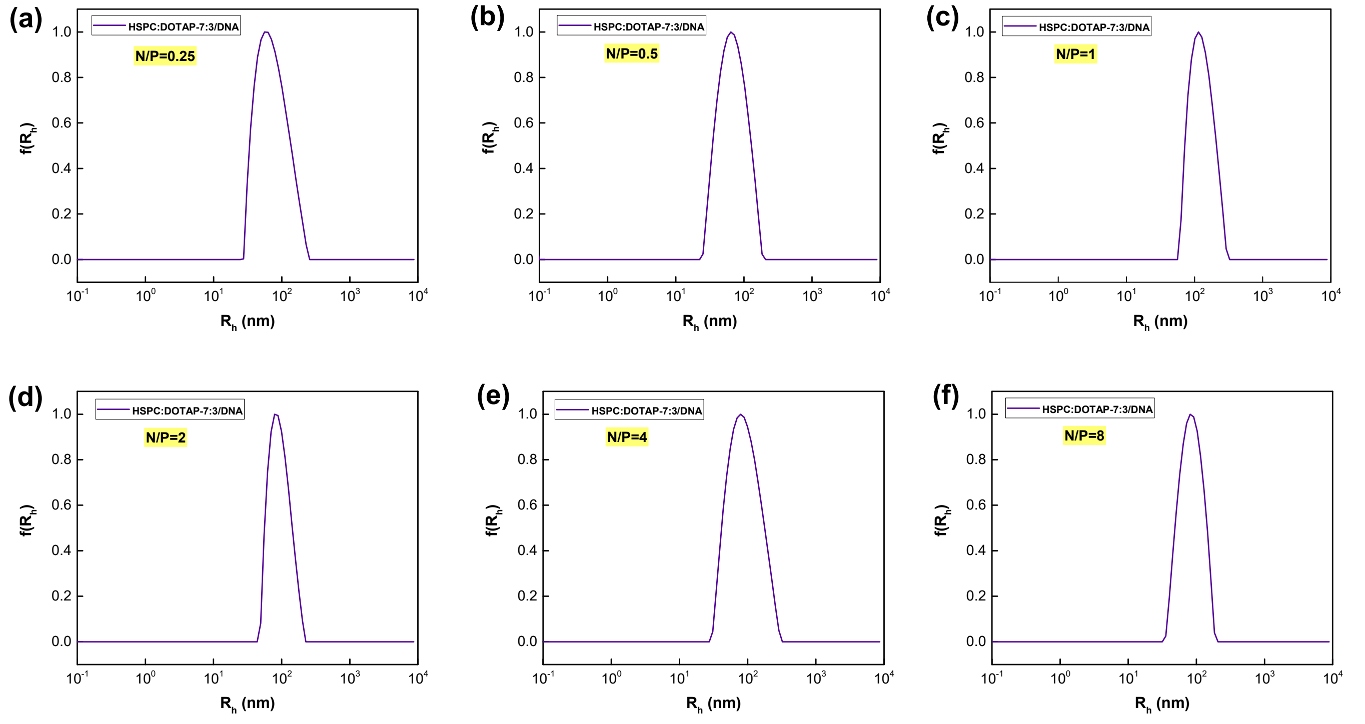

Figure S7. Size distributions from CONTIN analysis of HSPC:DOTAP-7:3/DNA lipoplexes, at ratios (**a**) N/P=0.25, (**b**) N/P=0.5, (**c**) N/P= 1 (partial precipitation takes place at N/P=1, data shown are from supernatant solution), (**d**) N/P=2, (**e**) N/P=4 and (**f**) N/P=8.

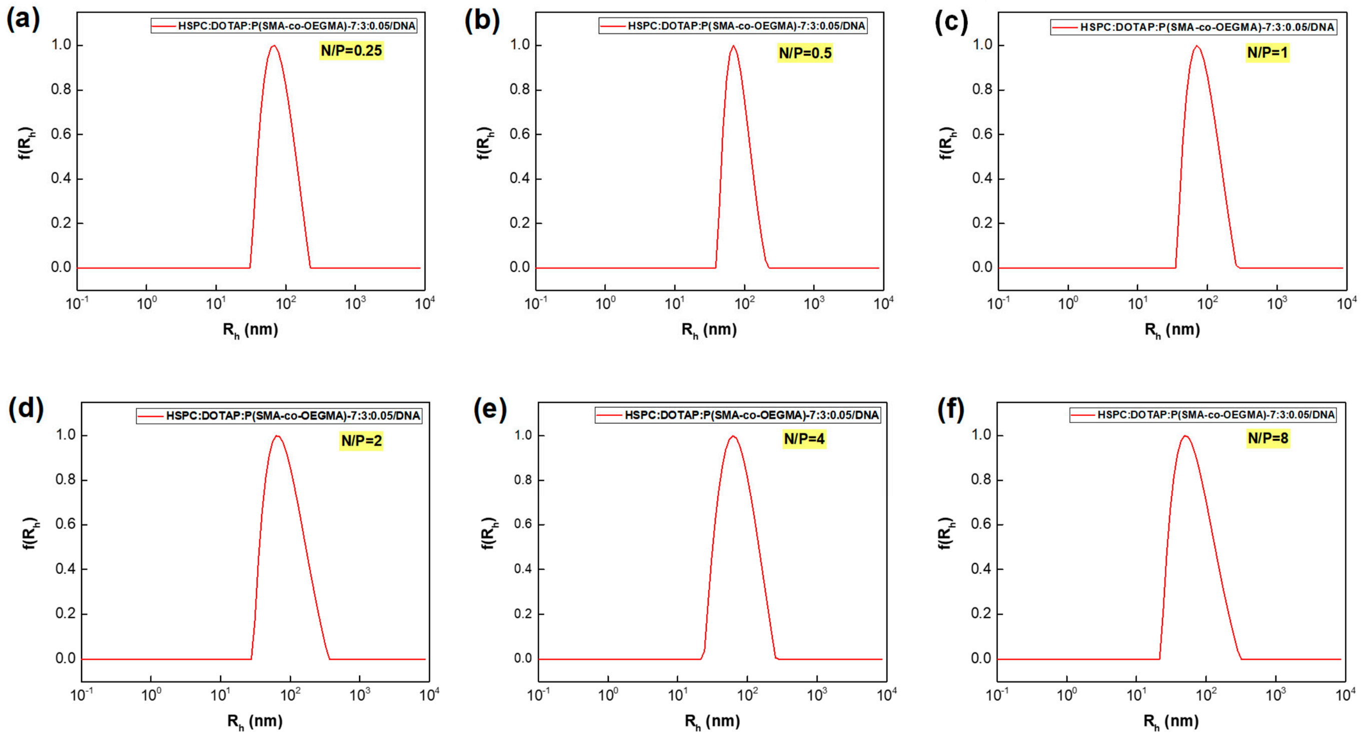

Figure S8. Size distributions from CONTIN analysis of HSPC:DOTAP-7:3:0.05/DNA chimeric lipoplexes, at ratios (**a**) N/P=0.25, (**b**) N/P=0.5, (**c**) N/P= 1 (partial precipitation takes place at N/P=1, data shown are from supernatant solution), (**d**) N/P=2, (**e**) N/P=4 and (**f**) N/P=8.

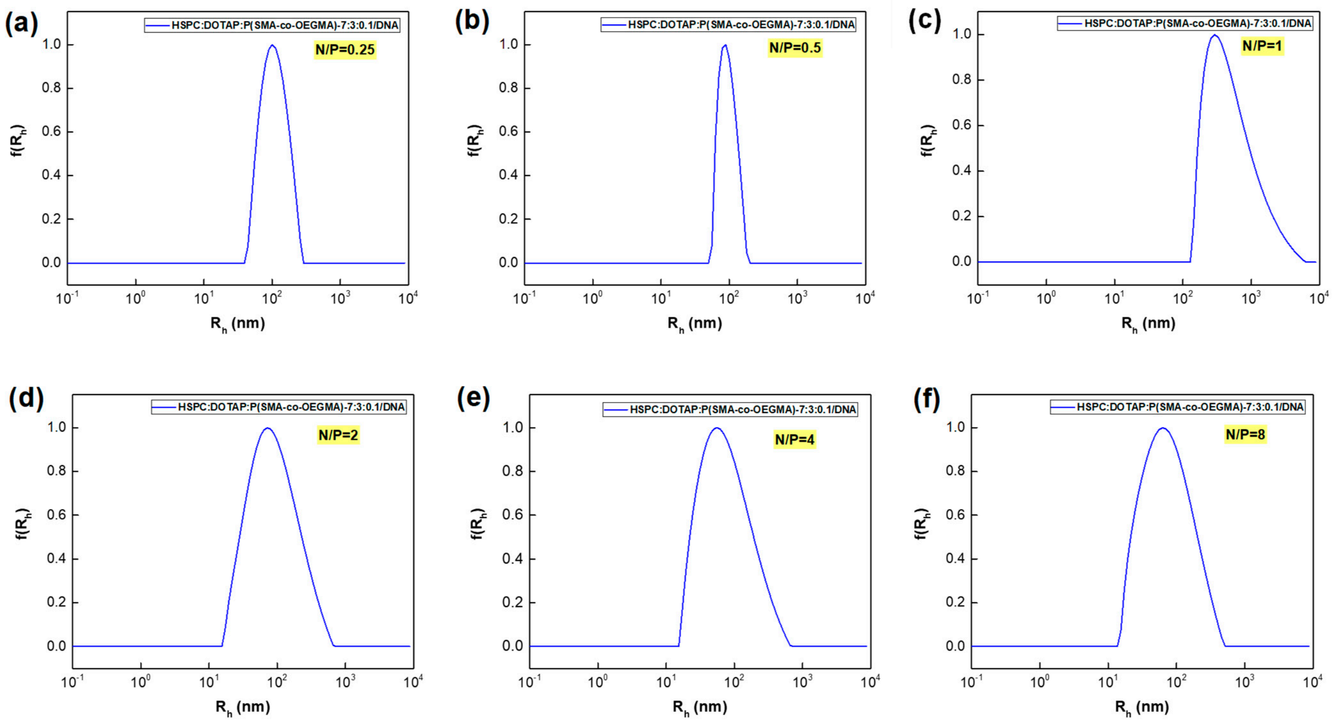

Figure S9. Size distributions from CONTIN analysis of HSPC:DOTAP-7:3:0.1/DNA chimeric lipoplexes, at ratios (**a**) N/P=0.25, (**b**) N/P=0.5, (**c**) N/P= 1 (partial precipitation takes place at N/P=1, data shown are from supernatant solution), (**d**) N/P=2, (**e**) N/P=4 and (**f**) N/P=8.

References

1. Chrysostomou, V.; Katifelis, H.; Gazouli, M.; Dimas, K.; Demetzos, C.; Pispas, S. Hydrophilic Random Cationic Copolymers as Polyplex-Formation Vectors for DNA. *Materials* **2022**, *15*, 2650. https://doi.org/10.3390/ma15072650.
2. Chrysostomou, V.; Forys, A.; Trzebicka, B.; Demetzos, C.; Pispas, S. Structure of micelleplexes formed between QPDMAEMA-b-PLMA amphiphilic cationic copolymer micelles and DNA of different lengths. *Eur. Polym. J.* **2022**, *166*, 111048. https://doi.org/10.1016/j.eurpolymj.2022.111048.
